# Conditional deletion of *Wnt5a* in myeloid osteoclast precursors results in low bone mass via disruption of osteoblast migration and focal adhesion dynamics

**DOI:** 10.3389/fcell.2026.1868995

**Published:** 2026-07-22

**Authors:** Valentina Daponte, Chandra Gavva, David J. Kapfhamer, Joseph Roberts, Katrin Henke, Hicham Drissi

**Affiliations:** 1 Department of Orthopaedics, School of Medicine, Emory University, Atlanta, GA, United States; 2 The Atlanta Veterans Affairs Health Care System, Decatur, GA, United States; 3 College of Health Solutions, Arizona State University, Phoenix, AZ, United States

**Keywords:** clastokine, coupling, osteoblasts, osteoclasts, Wnt5a

## Abstract

**Introduction:**

Bone remodeling depends on coordinated signaling between bone-resorbing osteoclasts and bone-forming osteoblasts through locally secreted coupling factors. Disruption of this balance leads to decreased bone mass, resulting in conditions such as osteoporosis. Osteoclast-derived anabolic factors, or clastokines, have emerged as attractive therapeutic candidates with the potential of selectively enhancing bone formation without suppressing resorption. Osteoblast-derived WNT5A is an established coupling factor promoting osteoclastogenesis; however, osteoclast-derived WNT5A specific contribution to bone homeostasis and the downstream mechanism by which it acts on osteoblasts remains undefined.

**Methods:**

This study aimed to address this gap using a myeloid-specific conditional knockout mouse that selectively targets osteoclast-derived WNT5A while leaving osteoblast-derived WNT5A intact. Wnt5a was conditionally deleted in osteoclast precursors and their progeny using LysM-Cre; Wnt5aF/F mice and both male and female knockout mice were characterized by μCT, histomorphometry, serum bone turnover markers, transcriptomic profiling of osteoblast-enriched cells, and in vitro focal adhesion and migration assays.

**Results:**

Both male and female knockout mice displayed significantly reduced trabecular volume compared with controls. This low bone mass phenotype was driven by decreased osteoblast number and activity, with no significant changes in osteoclast number or resorptive function. Transcriptomic profiling of osteoblast-enriched cells revealed dysregulation of genes involved in focal adhesion, integrin binding, and cell communication, alongside upregulation of stress-related gene networks. Histological and functional analyses demonstrated that loss of osteoclast-derived WNT5A impaired extracellular matrix maturation and disrupted osteoblast focal adhesion assembly and migration.

**Discussion:**

Together, our study establishes osteoclast-derived WNT5A as a clastokine that promotes bone formation in vivo by regulating osteoblast migration and adhesion dynamics. These findings establish a mechanistic link between osteoclast secretory activity and osteoblast function and highlight the context-dependent and cell-of-origin specific roles of WNT5A in skeletal homeostasis.

## 1 Introduction

Bone homeostasis is maintained by the tightly coordinated actions of bone-forming osteoblasts and bone-resorbing osteoclasts, which operate together within the basic multicellular unit (BMU) (Sims and Martin, 2014). Disruption of the balance between bone formation and resorption can compromise skeletal integrity, leading to increased fragility and fracture risk, as seen in conditions such as osteoporosis (Seeman, 2003). Current therapeutic strategies typically target either bone formation or resorption, often at the expense of the other, thereby disrupting homeostasis and causing unintended side effects (Carvalho et al., 2011; Koh et al., 2010; Khan et al., 2009). This highlights the need to identify factors that can selectively enhance osteoblast function without affecting osteoclast activity. In the past decade, increasing evidence has shed new light on the ability of osteoclasts to promote and direct bone formation by secreting anabolic molecules, also known as clastokines (Daponte et al., 2024), providing an opportunity to identify new therapeutic targets for bone diseases that preserve coupling.

Among the signaling molecules implicated in bone remodeling, WNT proteins have emerged as key regulators of skeletal maintenance by influencing osteoblast-osteoclast communication (Pederson et al., 2008; Maeda et al., 2012; Okamoto et al., 2014; Kobayashi et al., 2015; Hu et al., 2024). WNT5A is a versatile, non-canonical WNT protein that acts through several receptors, including Ror2 and Frizzled (Fzd) receptors (Maeda et al., 2012; Bolzoni et al., 2011). WNT5A is primarily secreted by osteoblasts and, to a lesser extent, by osteoclasts (Uehara et al., 2017). It plays key roles in skeletal development and limb morphogenesis by regulating progenitor cell proliferation during embryogenesis (Yamaguchi et al., 1999). Additionally, WNT5A has a well-established role in skeletal health: mutations in *WNT5A* cause autosomal dominant Robinow syndrome, characterized by short stature, mesomelic limb shortening, hypertelorism, mandibular hypoplasia, and irregular dental alignment (Robinow et al., 1969; Person et al., 2010; Mazzeu et al., 2007). Previous work has shown that osteoblast-secreted WNT5A enhances osteoclastogenesis by inducing RANK expression and abrogating the inhibitory effects of WNT16 on RANKL-RANK signaling in osteoclast precursors (Maeda et al., 2012; Kobayashi et al., 2015). Osteoblast-derived WNT5A has also been shown to promote osteoblast differentiation through several autocrine mechanisms, including modulation of noncanonical and canonical WNT signaling pathways and cooperation with BMP2-mediated osteoblastogenesis (Okamoto et al., 2014; Nemoto et al., 2012; Guo et al., 2008). More recent studies have added further complexity. In osteoporotic adipose-derived stem cells, an inverse relationship between non-canonical WNT5A and osteogenesis has been reported, in which silencing WNT5A enhanced osteogenic differentiation and bone repair in critical-size defect models, possibly through de-repression of canonical pathways (Liu et al., 2025). Together, these studies have shown that the effects of WNT5A are strongly cell- and context-dependent.

WNT5A is also expressed by osteoclasts, although its role remains poorly defined (Uehara et al., 2017). In previous work, we employed the *Ctsk-Cre; Wnt5a* ^*F/F*^ mouse model to delete *Wnt5a* from mature osteoclasts (Roberts et al., 2020). Surprisingly, male mice exhibited reduced bone mass due to impaired bone formation, with no changes in bone resorption, suggesting an anabolic function for osteoclast-derived WNT5A (Roberts et al., 2020). However, the use of the *Ctsk-Cre* line to determine the role of osteoclast-derived WNT5A had several limitations, as *Ctsk* is also expressed in periosteal and other mesenchymal cells (Debnath et al., 2018; Zou et al., 2022). In addition, *Ctsk* expression is inhibited by estrogen, likely affecting Cre-mediated WNT5A transgene recombination efficiency in females, explaining the lack of a skeletal phenotype in female mice (Roberts et al., 2020; Troen, 2006).

To overcome these limitations and more directly define the contribution of osteoclast-derived WNT5A to bone remodeling, here we employed *LysM-Cre* to delete *Wnt5a* in myeloid lineage cells including osteoclast precursors (Clausen et al., 1999; Dallas et al., 2018). This complementary approach enabled us to more directly define the osteoclast-lineage effects of WNT5A function while excluding periosteal and mesenchymal cell contributions and allowing analysis in both sexes.

We found that male and female *LysM-Cre; Wnt5a* ^*F/F*^ mice exhibit reduced bone mass due to reduced osteoblast number and activity, with no changes in bone resorption. This phenotype mirrors that observed in our previous *Ctsk-Cre* model, providing independent genetic evidence supporting osteoclast-derived WNT5A as a clastokine, while extending the observation to female mice. To add to our previous study, here we investigated the previously unexplored mechanistic basis of osteoclast-derived WNT5A in bone remodeling. By employing RNA-seq analysis of osteoblast-enriched cells, we identified a downregulation of ECM-receptor interaction, Integrin signaling, Focal adhesion, and PI3K-Akt signaling pathways. Consistent with these transcriptional changes, functional assays demonstrated that loss of osteoclast-derived WNT5A impairs osteoblast focal adhesion assembly and migration *in vitro*. Histological analyses further revealed defective extracellular matrix (ECM) maturation. While WNT5A-derived regulation of focal adhesion dynamics and cell migration has been described in non-skeletal contexts, including melanoma (Weeraratna et al., 2002; Dissanayake et al., 2007), gastric carcinoma (Kurayoshi et al., 2006), and most recently Ewing sarcoma (Baker et al., 2025), this mechanism had not been previously explored in the context of osteoclast-osteoblast paracrine signaling. The present study provides the first evidence linking osteoclast-derived WNT5A to osteoblast focal adhesion assembly and integrin-ECM interactions, identifying a novel downstream effector axis underlying osteoclast-osteoblast coupling that is distinct from the WNT5A/ROR2 and planar cell polarity pathways previously described in bone.

Taken together, our findings expand the current WNT5A paradigm, establishing a model in which osteoblast-derived WNT5A promotes bone resorption, whereas osteoclast-derived WNT5A supports bone formation. This novel concept highlights the cell-context-dependent role of WNT5A and provides an important foundation for exploring WNT5A-targeted strategies to enhance bone anabolism without the risk of increasing resorptive activity.

## 2 Materials and methods

### 2.1 Animals

*LysM*-*Cre* (IMSR_JAX:004781) and *Wnt5a* floxed (*Wnt5a* ^F/F^, IMSR_JAX:026626) mice were from Jackson Laboratories. *LysM-Cre* mice are characterized by the insertion of Cre cDNA in the translational start site of the endogenous Lysozyme M gene and are well-characterized for specific expression and high deletion efficiency in myeloid cells, including osteoclast precursors (Clausen et al., 1999; Dallas et al., 2018).

Conditional knockout mice (*LysM-Cre; Wnt5a* ^*F/F*^, referred to as *Wnt5a* cKO) were compared with *Wnt5a* ^F/+^ littermate controls. All mice were maintained on a C57BL/6J background throughout this study. All experiments were performed on 10-week-old animals. Mice were housed at the Atlanta Veterans Affairs Medical Center (VAMC) animal facility under controlled conditions (temperature, 21 °C–24 °C; humidity, 40%–70%; light/dark cycle, 12/12 h) with free access to food and water. Mice were maintained following applicable state and federal guidelines. All experimental procedures were approved by the Atlanta VAMC Institutional Animal Care and Use Committee (IACUC).

### 2.2 Bone marrow macrophage (BMM) isolation and culture

Whole bone marrows from *Wnt5a* cKO and control mice were isolated from pooled femurs and tibiae by flushing the medullary cavity with α-MEM medium (Gibco). Red blood cells were removed using RBC Lysis Buffer (Invitrogen) and the remaining cells were expanded in the presence of recombinant murine M-CSF (30 ng/mL) for 2 days.

### 2.3 Osteoclastogenesis assay

Expanded bone marrow macrophages (BMMs) were seeded for experiments in 96-well plate and cultured in α-MEM medium (Gibco), supplemented with 10% FBS (Gibco), 1% antibiotic-antimycotic (Gibco), and either 30 ng/mL recombinant murine M-CSF alone or recombinant murine M-CSF (30 ng/mL) and 50 ng/mL recombinant murine sRANKL (Peprotech) to induce differentiation into osteoclasts. The cells were stained with TRAP (Kamiya Biomedical Company) to quantify the number of TRAP + mature osteoclasts (≥3 nuclei) per well after 3 and 5 days of culture. No distinction was made between large and small osteoclasts.

### 2.4 Pit assay

Pit assay was performed according to a published protocol (Cen et al., 2022). Briefly, expanded bone marrow macrophages (BMMs) were seeded on calcium-phosphate-coated 96-well plates in α-MEM medium (Gibco), supplemented with 10% FBS (Gibco), 1% antibiotic-antimycotic (Gibco), and either 20 ng/mL recombinant murine M-CSF alone or recombinant murine M-CSF (20 ng/mL) and 20 ng/mL recombinant murine sRANKL (Peprotech). At 9 days of culture, total resorption pit areas in each well were quantified after 5% AgNO_3_ staining and scanning with a Celigo Cytometer (Nexcelom Bioscience). Three wells per biological replicate were used for each experiment.

### 2.5 RNA isolation from BMM-derived osteoclasts and qPCR

Total RNA was isolated from BMM-derived, RANKL-stimulated osteoclasts at day 5 of culture using TRIzol (Invitrogen) according to the manufacturer’s instructions. Reverse-transcription was performed on 1 µg of RNA using the High-Capacity cDNA Reverse Transcription Kit (Applied Biosystems) in a final volume of 20 µL. Real time quantitative PCR (qPCR) was used to evaluate gene expression of *Wnt5a* and osteoclast markers *Ctsk*, *TRAP*, and *Calcr* in control and *Wnt5a* cKO cells. *β-Actin* was used as a reference gene (Supplementary Table S1). qPCR was performed on Rotor-Gene Q (Qiagen) using SYBR Green Master mix (Applied Biosystems). Amplicon authenticity was confirmed by melting curve analysis. The relative expression was calculated using ΔΔCt method.

### 2.6 Micro-computed tomography (µCT)

Micro-computed tomography (µCT) was performed on the femur and 3rd lumbar (L3) vertebra *ex vivo* to assess trabecular and cortical bone microarchitecture using a µCT40 scanner (Scanco Medical AG) that was calibrated weekly using a factory-supplied phantom. Bones were first fixed for 1 week in 10% neutral buffered formalin at 4 °C, followed by scanning in phosphate-buffered saline (PBS). Femur trabecular bone indices were determined from a total of 100 tomographic slices that were taken from the distal femoral metaphysis, starting 0.5 mm proximal from the distal growth plate at a voxel size of 6 µm (70 kVp and 114 mA, with 200 ms integration time). For L3 vertebrae, projection images were reconstructed using the auto-contour function for vertebral body trabecular bone between the cranial and caudal growth plates from approximately 350 tomographic slices at a voxel size of 6 µm (70 kVp and 114 mA, with 200 ms integration time). Trabecular bone representative samples were reconstructed in 3D to generate visual representations based on mean BV/TV. The following 3D indices in the defined region of interest (ROI) were analyzed: bone volume fraction (BV/TV, %), trabecular number (Tb.N, mm^−1^), trabecular thickness (Tb.Th, mm). All indices and units were standardized according to published guidelines (Bouxsein et al., 2010).

### 2.7 Histology and histomorphometry

Femurs were fixed for 1 week in 10% neutral buffered formalin at 4 °C and then decalcified in 14% EDTA (pH 7.2) for 2 weeks before embedding in paraffin. 5 μm–thick sections were obtained and stained for Hematoxylin and Eosin (H&E) or Tartrate-resistant acid phosphatase (TRAP) (Kamiya Biomedical Company). Three sections in the sagittal plane from each bone were analyzed using a 600 µm-wide ROI beginning 200 µm distal to the growth plate. The following indices were analyzed on H&E-stained sections: bone volume fraction (BV/TV, %), trabecular number (Tb.N, mm^−1^), trabecular thickness (Tb.Th, µm) and trabecular spacing (Tb.Sp, µm). Additionally, the number of osteoblasts per bone perimeter (N.Ob/B.Pm, mm^-1^) and the number of osteoblasts per tissue area (N.Ob/T.Ar,/mm^2^) were counted. Osteoblasts were identified as groups of cuboidal cells located around bone trabeculae. Flat bone lining cells, not reflective of active bone formation, were excluded from the counting. On TRAP-stained sections, the number of TRAP + osteoclasts per bone perimeter (N.Oc/B.Pm, mm^−1^) and osteoclast surface per bone surface (Oc.S/BS, %) were determined. All histomorphometric measurements were performed according to the criteria established by the American Society of Bone and Mineral Research using the Osteomeasure software (Osteometrics) connected to an Olympus BX53 microscope (Olympus) (Dempster et al., 2013). Representative images were acquired using an Olympus BX63 microscope (Olympus).

### 2.8 Bone turnover marker measurement

Mice were fasted without food but with free access to water for at least 6 h before sacrifice. Whole blood was obtained by cardiac puncture at the time of sacrifice, allowed to clot at room temperature for 30 min, and then centrifuged at 10,000 x *g* for 10 min. Serum was removed, aliquoted, and stored at −80 °C. C-terminal collagen cross-links (CTX) (RatLaps; ImmunoDiagnostic Systems #AC-06F1) and N-terminal propeptide of type I procollagen (P1NP) (ImmunoDiagnostic Systems #AC-33F1) were assayed using enzyme-linked immunosorbent assay kits according to the manufacturer’s recommendations.

### 2.9 Torsional testing

Femurs were collected from animals, carefully dissected free of all soft tissue, and stored in phosphate-buffered saline-soaked gauze at −20 °C prior to testing. Proximal and distal ends of femurs were aligned and securely potted into 1 cm^2^ metal cubes filled with polymethylmethacrylate (PMMA) bone cement. Torsion was applied at a rate of 0.8 deg/sec to the proximal end of the bone using an electromechanical testing apparatus equipped with a torsion add-on (68SC-2, Torsion Add-On 3.0, Instron Corp.) until yield. Maximum torque and angle at failure were recorded for all samples and used to calculate torsional rigidity and work to failure.

### 2.10 Endosteal cells isolation

Endosteal bone cells were isolated from long bone endocortical surfaces of male mice following a published method (Ayturk et al., 2020). These cells represent a mixed population of diverse cell types, including a subset with transcriptional profiles consistent with mesenchymal and osteoblast lineage cells. Briefly, femurs and tibias were collected, distal and proximal epiphyses were cut away with a scalpel, and bone marrow was removed by flushing with α-MEM medium (Gibco). The periosteum was aseptically removed using a scalpel and bones were bisected lengthwise to expose the endosteum. The long bones from each mouse were combined and incubated with 4 mL of collagenase solution (3 mg/mL Collagenase Type II in PBS, Gibco) at 37 °C for 15 min under continuous agitation. The first digestion was discarded, and bones were digested in two additional 4 mL collagenase solutions for 30 min each. Cells recovered with each digestion were mixed with an equal volume of α-MEM (Gibco) supplemented with 10% FBS (Gibco), 1x antibiotic-antimycotic (Gibco). Cells were pelleted at 2000 x *g* for 5 min and used for RNA isolation. To verify that collagenase digestion efficiently removed cells from the endosteal bone surface, bones were processed for histology as described above, stained with H&E, and compared to similar sections from bones that had been placed in PBS (Supplementary Figure S1).

### 2.11 RNA isolation from pelleted endosteal cells

RNA was isolated using the RNeasy Kit (Qiagen) following manufacturer’s instructions. The purification included a DNase treatment using the RNase free DNase Set (Qiagen). RNA concentration was determined by NanoDrop spectrophotometer. RNA integrity was determined using Agilent 2200 Bioanalyzer. Samples with a 260/280 ratio >1.8 and an RNA Integrity Number (RIN) > 7 were selected for sequencing.

### 2.12 Library preparation and bulk RNA-sequencing

Library preparation and ultra-low input sequencing were performed at Admera Health. Libraries were prepared from a minimum of 35 ng of RNA using NEBNext Ultra II Directional RNA Library Prep Kit (New England Biosciences) using PolyA Selection. Next-Generation Sequencing was carried out by NovaSeq 6000 (Illumina) using 2 × 150 bp paired-end reads, generating approximately 40 million reads per sample (20 million reads per direction).

### 2.13 Differential gene expression analysis

Raw data were exported in FASTQ (fq) format and FastQC (v0.11.8) was applied to check error rate and GC content distribution (Andrews, 2010). Trimmomatic (v0.38) was used to cut adaptors and trim low-quality bases with default settings (Bolger et al., 2014). Trimmed reads were aligned to the *Mus musculus* GRCm38 reference genome using DNASTAR SeqMan Ngen. Unique gene hit counts were calculated using HTseq-count (version 0.11.2) (Anders et al., 2015). The gene hit counts table was used for downstream differential expression analysis. Genes highly expressed in potential contaminant tissues, such as skeletal muscle, blood, and bone marrow, were identified based on the Ayturk et al. (2013) RNA-seq study and were filtered from the gene hit count list prior to downstream analysis to reduce non-skeletal signal (Ayturk et al., 2013). Read counts were analyzed using iDEP v2.4.3 (Ge et al., 2018). Genes with counts below 0.5 CPM in at least 5 samples were removed, and the remaining data were transformed using EdgeR (Robinson et al., 2010). Differential expression analysis was performed using the DESeq2 option on the iDEP website (Ge et al., 2018; Love et al., 2014). A false discovery rate (FDR) cutoff of 0.05 and an absolute fold-change threshold ≥1.5 were set for statistically significant DEGs.

### 2.14 Pathway enrichment analysis

Gene Ontology (GO) analysis was performed to identify enriched biological processes and molecular functions. GO analysis was conducted using Gorilla (Eden et al., 2009), and redundant terms were removed from GO Biological Process using REVIGO (Supek et al., 2011). Significant GO terms were visualized by dotplot using ggplot2 in R. GeneRatio was calculated by the ratio of gene counts enriched in a particular GO term to all the gene counts annotated in that GO term.

GSEA was conducted based on KEGG (Kyoto Encyclopedia of Genes and Genomes) database using the clusterProfiler package in R (Wu et al., 2021). A gene set was considered significantly enriched when the false discovery rate (FDR) was less than 0.05. Top 10–15 leading-edge genes for each enriched pathway, ranked based on logFC, were used to generate chord diagram using the GOplot function in R.

### 2.15 Picro-sirius red staining

Staining was performed on 5 µm-thick, paraffin sections using the Picro-Sirius Red Stain Kit (#KT 037-IFU, Diagnostic BioSystems) following the manufacturer’s instructions. Birefringent images were acquired using an Olympus BX63 microscope (Olympus) equipped with a polarizer. Quantification of Red-Orange, Yellow, and Green areas was performed on a 600 µm-wide ROI below the growth plate, including trabecular and cortical bone, using the Picrosirius Red Birefringence Analyser Plugin on Fiji (ImageJ) (Vennin et al., 2019).

### 2.16 Conditioned medium (cM) from RANKL-differentiated BMMs

BMMs were differentiated into osteoclasts from male control and *Wnt5a* cKO mice as described above. Mature osteoclasts were then cultured for 48 h without FBS to exclude the contribution of residual serum components. Media was collected and concentrated using 10 kDa cutoff Amicon filters (Millipore) and centrifuged at 3,000 rpm for 45 min to obtain conditioned medium (cM), following a previously published approach in which WNT5A protein was successfully detected under equivalent conditions (Roberts et al., 2020).

### 2.17 MC3T3-E1 focal adhesion assay

Osteoblastic MC3T3-E1 cells were obtained from ATCC and cultured in α-MEM medium (Gibco), supplemented with 10% FBS (Gibco) and 1% Pen/Strep (Gibco). 1 × 10^4^ cells were seeded on glass coverslips coated with 5 μg/cm^2^ collagen I (Gibco) in 6-well plates. After cell adhesion, medium was switched to 30% cM from control or *Wnt5a* cKO (n = 3 biological replicates), and cells were cultured for an additional 24 h. Cells were fixed in 10% neutral buffered formalin, permeabilized with 0.1% Triton X-100, and incubated with Phalloidin-Alexa Fluor 488 (Thermo Fisher A12379) and anti-Paxillin (Cell Signaling 50195, 1:800) in 1% Bovine Serum Albumin (BSA) overnight. After that, cells were stained with DAPI for 10 min. Samples were imaged using a Leica Stellaris confocal microscope equipped with a 63x oil immersion objective. Images were acquired in a blinded manner. Fields were selected randomly across the slide prior to unblinding. All acquisition parameters were kept constant across samples within each experiment. The number and total area of focal adhesion per cell were quantified using ImageJ software. Briefly, single z-slices were converted to 8-bit and calibrated using microscope metadata. Paxillin images were denoised with a median filter (radius = 1 px), and background was gently corrected using rolling-ball subtraction (40 px). Focal adhesions were segmented by Triangle thresholding and converted to binary images. Particles ≥0.25 µm^2^ were quantified. Cell area was independently segmented from Phalloidin staining using Gaussian smoothing and Triangle thresholding, and focal adhesion analysis was restricted to the corresponding cell ROI. Negative controls are reported in Supplementary Figure S2.

### 2.18 MC3T3-E1 wound healing assay

*In vitro* wound healing assay was carried out by CytoSelect™ 24-Well Wound Healing Assay according to manufacturer’s instructions. Osteoblastic MC3T3-E1 cells were cultured in α-MEM medium (Gibco), supplemented with 10% FBS (Gibco) and 1% Pen/Strep (Gibco). 5 × 10^4^ cells were seeded on 24-well plates containing kit inserts and incubated until reaching 80% confluency. The inserts were removed from the wells, and medium was switched to 30% cM from control or *Wnt5a* cKO (n = 3 biological replicates). Cell migration percentage was evaluated after 24 h using the following formula (Area 0h–Area 24h)/Area 0h × 100.

### 2.19 Statistical analysis

All values are presented as mean ± standard deviation (SD). Statistical analysis of data was determined by unpaired two-tailed Student’s t-test, Mann-Whitney, or ANOVA followed by Tukey’s *post hoc* test, as indicated in figure legends using GraphPad Prism (v10.2.0) software. Normality was tested through the Shapiro-Wilk test. A p-value <0.05 was considered statistically significant.

## 3 Results

### 3.1 Conditional deletion of *Wnt5a* in myeloid osteoclast precursor cells

**FIGURE 1:**
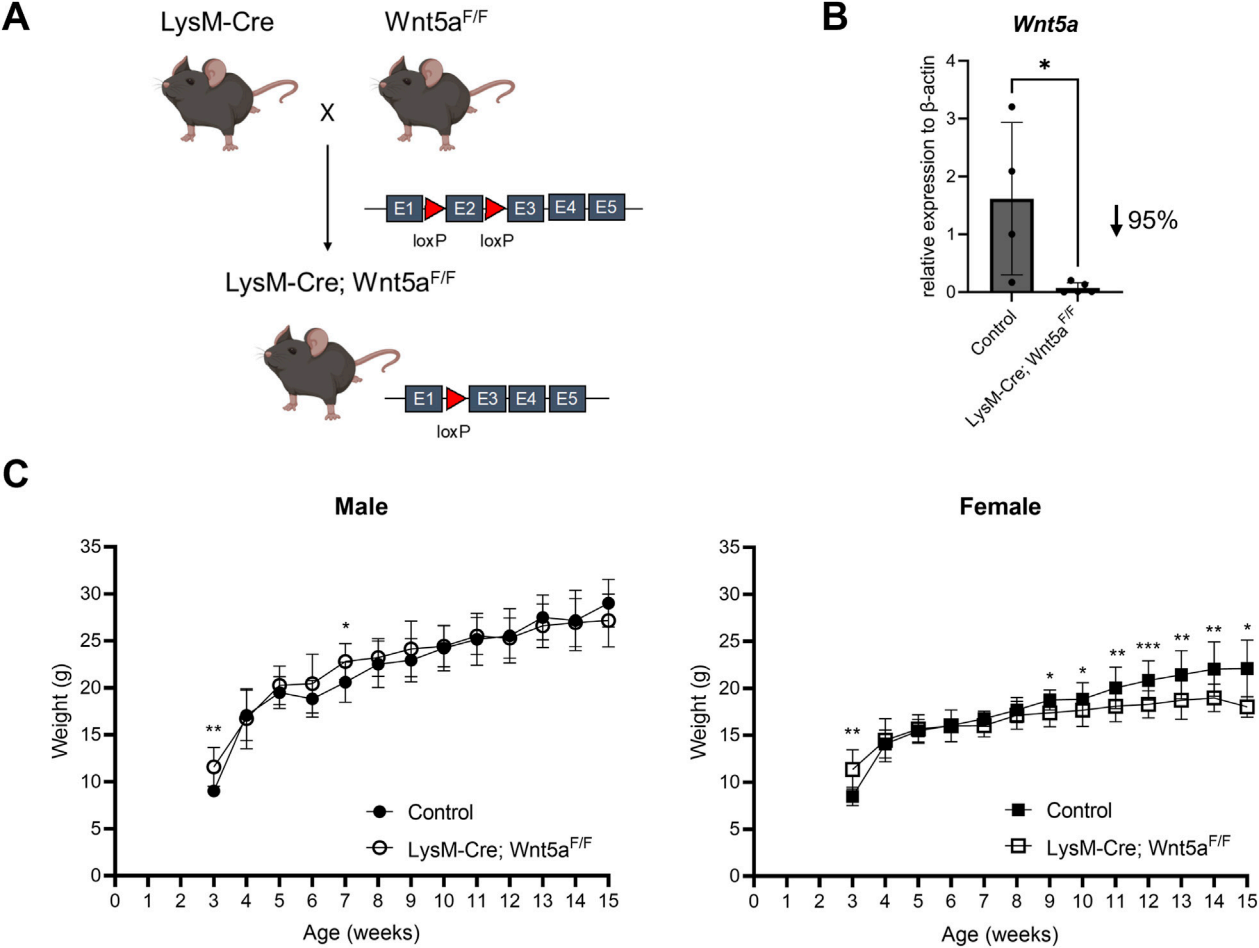
Generation of conditional *Wnt5a* loss-of-function model in osteoclast precursors. **(A)** *LysM-Cre:Wnt5a* ^*F/F*^ (*Wnt5a* cKO) mice were generated by crossing *LysM-Cre* mice to *Wnt5a* ^*F/F*^ mice. Cre-mediated recombination in osteoclast precursors results in the excision of Exon 2 of the *Wnt5a* gene. **(B)** Gene expression analysis by qPCR using primers flanking the excised Exon 2 shows significant reduction of *Wnt5a* expression in BMMs treated with RANKL for 5 days, confirming successful targeting of the gene in osteoclasts (Control n = 4; *Wnt5a* cKO n = 5). Statistical analysis was performed using Mann-Whitney U Test. *p < 0.05. **(C)** Body weight of male and female animals from weaning (3 weeks) to 15 weeks, including the experimental time of 10 weeks. Female *Wnt5a* cKO show consistent reduction in body weight compared to controls starting at 9 weeks of age (males n = 6–24/genotype; females n = 3–30/genotype). Statistical analysis was performed by unpaired, two-tailed *t*-test by comparing different genotypes of the same age. *p < 0.05, **p < 0.01, ***p < 0.001.

To conditionally knockout *Wnt5a* in myeloid cells, *Wnt5a* ^F/F^ mice were crossed to mice harboring the *Cre* recombinase gene in the native lysozyme 2 (*Lyz2*) gene locus, encoding for Lysozyme M (*LysM*) (*LysM-Cre*), a well-characterized line commonly used to target osteoclast precursors (Figure 1A) (Clausen et al., 1999; Dallas et al., 2018). We generated male and female mice lacking *Wnt5a* in *LysM-*expressing cells (*LysM-Cre; Wnt5a* ^*F/F*^), hereafter referred to as *Wnt5a* cKO, in which Cre-mediated recombination in osteoclast precursors leads to excision of exon 2 of the *Wnt5a* gene. To assess the efficiency of *Wnt5a* recombination in osteoclasts, bone marrow macrophages (BMMs) were isolated from the long bones of 10-week-old *Wnt5a* cKO and control mice and differentiated into osteoclasts by RANKL stimulation for 5 days. *Wnt5a* mRNA levels decreased by 95% in *Wnt5a* cKO cells compared to control cells, confirming successful gene deletion (Figure 1B).

*Wnt5a* cKO mice were viable, born at expected Mendelian ratios, and their gross morphology was indistinguishable from controls. Body weight measured from weaning (3 weeks) to early adulthood (15 weeks), including the experimental age of 10 weeks, revealed that after 9 weeks of age female *Wnt5a* cKO mice had consistently lower body weight compared to littermate controls (Figure 1C). In contrast, male *Wnt5a* cKO body weight remained comparable to controls, suggesting a potential sex-dimorphic metabolic effect of the deletion.

### 3.2 Deletion of *Wnt5a* in osteoclast precursors does not affect osteoclast activity *in vitro*

**FIGURE 2:**
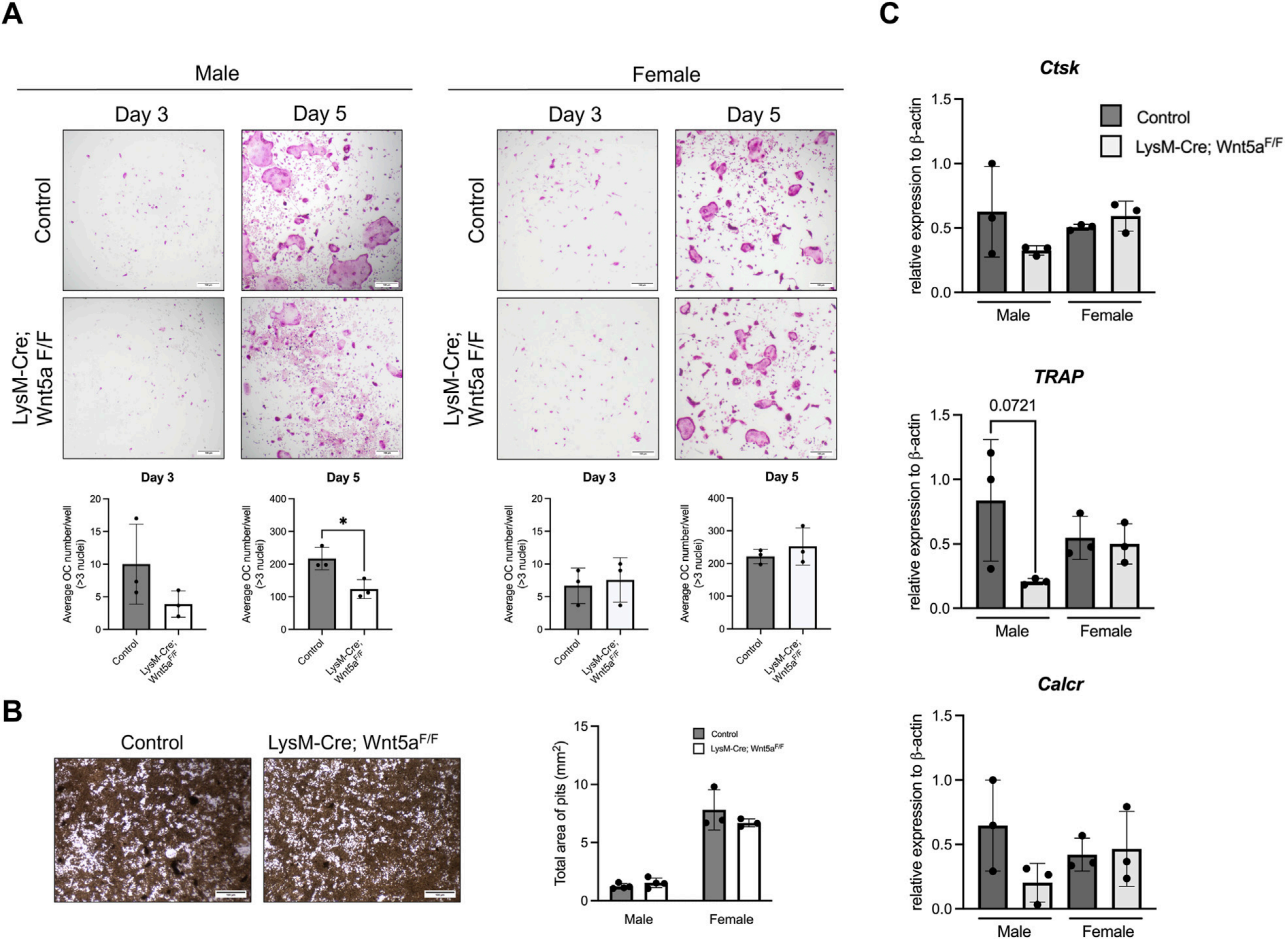
Conditional deletion of *Wnt5a* in osteoclast precursors does not affect osteoclast function *in vitro*. **(A)** The number of TRAP + osteoclasts (≥3 nuclei) is not affected at day 3 in males or females. At day 5, male *Wnt5a* cKO display significantly less TRAP + osteoclasts compared to controls (males n = 3/genotype; females n = 3/genotype). Statistical analysis was performed by unpaired, two-tailed *t*-test. *p < 0.05. **(B)** Bone resorption is not affected in either male or female *Wnt5a* cKO following 9 days of culture (males n = 4/genotype; females n = 3/genotype). Statistical analysis was performed by unpaired, two-tailed *t*-test. Representative image from females. **(C)** The gene expression levels of the osteoclastic genes *Ctsk*, *TRAP*, and *Calcr* were not affected by *Wnt5a* cKO mutation (males n = 3/genotype; females n = 3/genotype). Statistical analysis was performed by unpaired, two-tailed *t*-test.

Osteoblast-derived WNT5A has a well-established role in promoting osteoclastogenesis by upregulating RANK in osteoclast precursors (Maeda et al., 2012). To determine if osteoclast-derived WNT5A has a similar autocrine role, BMMs were isolated from the long bones of 10-week-old male and female *Wnt5a* cKO and control mice and induced to differentiate into osteoclasts in the presence of RANKL for 3 and 5 days. Conditional deletion of *Wnt5a* in myeloid cells did not influence *in vitro* osteoclast differentiation or function in female mice at either time point (Figure 2A). However, osteoclastogenesis was significantly reduced in male *Wnt5a* cKO compared to controls after 5 days of RANKL administration. Expression levels of the osteoclast genes *Ctsk* and *calcitonin receptor* (*Calcr*) did not differ between genotypes in osteoclasts at day 5 of culture, independent of sex (Figure 2B). The *TRAP* marker showed a slight, non-significant downward trend in male *Wnt5a* cKO. We next assessed the functional consequences of Wnt5a deletion on bone resorption *in vitro* using a pit assay. Deletion of *Wnt5a* did not change total pit area compared to controls in either sex (Figure 2C).

Taken together, our *in vitro* analyses suggest that *Wnt5a* deletion in myeloid cells does not affect osteoclast differentiation in females and, although it decreased osteoclast number in males, it did not alter osteoclast resorptive activity in either sex.

### 3.3 Decreased bone mass in male and female mice lacking *Wnt5a* in osteoclast precursors

**FIGURE 3:**
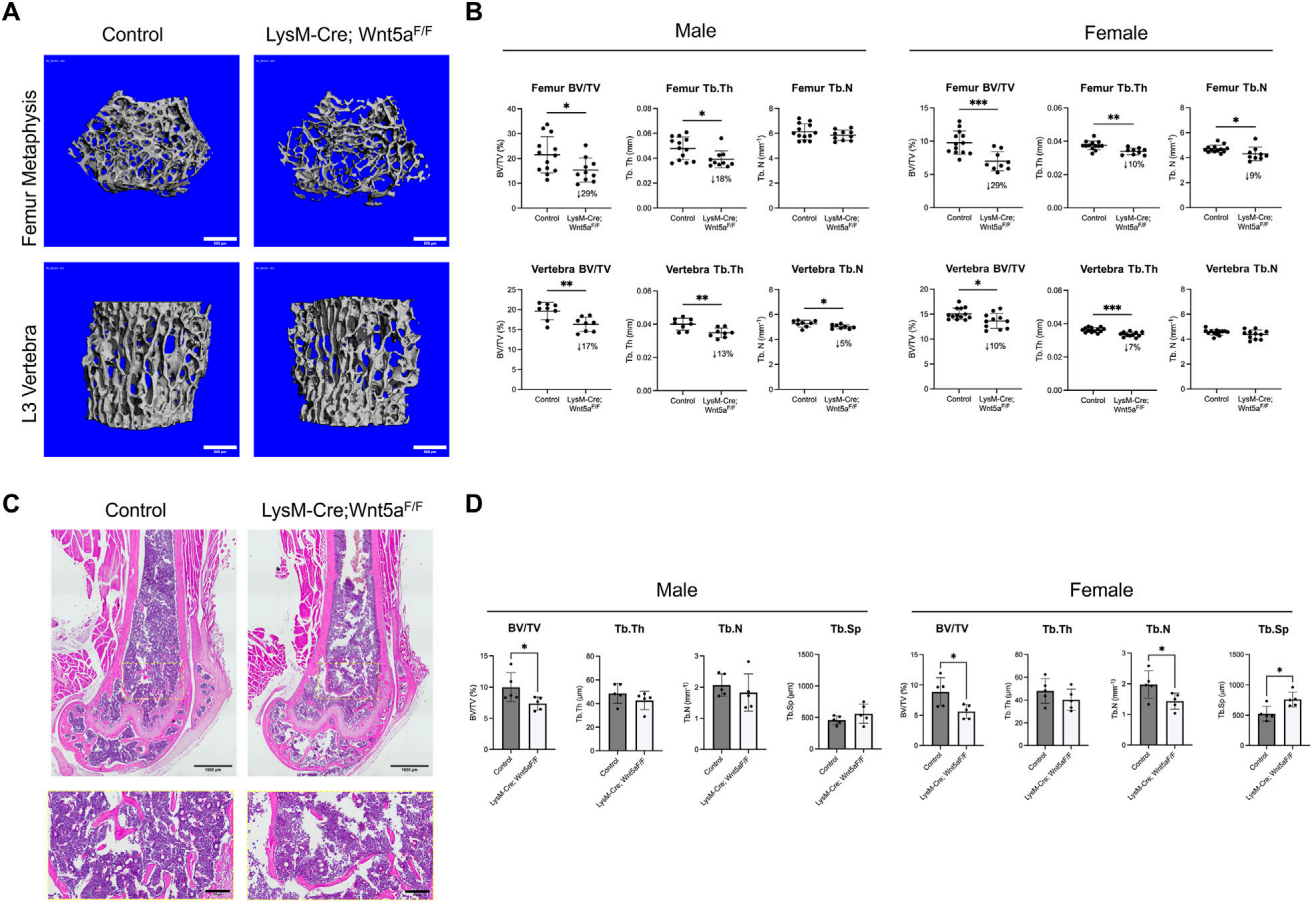
Conditional deletion of Wnt5a in osteoclast precursors disrupts bone growth, resulting in reduced bone mass. **(A,B)** Representative μCT 3D reconstructions of the trabecular bone within the distal femur metaphysis and the L3 vertebral body. μCT analyses revealed significantly reduced trabecular microarchitecture parameters in male and female Wnt5a cKO compared to their respective controls in both compartments (males n = 8-13/genotype; females n = 8-13/genotype). Statistical analysis was performed by unpaired, two-tailed t-test. * p < 0.05, ** p < 0.01, *** p < 0.001. **(C,D)** Representative section of female femur distal metaphysis section, stained with hematoxylin & eosin (H&E). Static histomorphometry of trabecular bone confirms the reduction in BV/TV in male and female animals (males n = 5/genotype; females n = 5/genotype). Statistical analysis was performed by unpaired, two-tailed t-test. * p < 0.05. BV/TV, bone volume fraction; Tb.Th, trabecular thickness; Tb.N, trabecular number; Tb.Sp, trabecular separation.

We next examined the microarchitecture of trabecular bone in femurs and L3 vertebral bodies to determine the effect of *Wnt5a* deletion in osteoclast precursors on bone mass. µCT analyses of the distal femoral metaphysis revealed a significant decrease in femoral trabecular bone volume fraction (BV/TV) in male (−29%) and female (−29%) *Wnt5a* cKO mice compared to controls (Figures 3A,B). Additionally, femoral trabecular thickness (Tb.Th) was reduced in males (−18%) and females (−10%). Femoral trabecular number (Tb.N) was decreased only in the female group (−9%). A similar osteopenic phenotype was observed in the trabecular microarchitecture of L3 vertebra, with male and female *Wnt5a* cKO presenting significantly reduced BV/TV (−17% and −10%, respectively) and Tb.Th (−13% and −7%, respectively), whereas Tb.N was significantly decreased only in males (−5%) compared to controls. No differences were observed in cortical bone (data not shown). Validating our µCT findings, static histomorphometry of the distal femur showed a significant decrease in BV/TV in both male and female *Wnt5a* cKO mice (Figures 3C,D). Tb.Th and Tb.N were also significantly reduced in female mutants, together with an increase in trabecular separation (Tb.Sp). However, these parameters did not reach statistical significance in males. Finally, to determine whether the impaired bone microarchitecture found in *Wnt5a* cKO mice would translate into changes in bone strength, we analyzed bone biomechanical properties of femora by *ex vivo* torsional testing. Male *Wnt5a* cKO displayed significantly reduced maximum torque and work to failure compared to controls, indicating compromised bone quality, whereas torsional rigidity remained unchanged (Supplementary Figure S3A). In contrast, no differences in femoral biomechanics were observed in female mice (Supplementary Figure S3B).

### 3.4 Decreased bone formation, with no changes in bone resorption *in vivo*, contributes to the osteopenic phenotype in *Wnt5a* cKO

**FIGURE 4:**
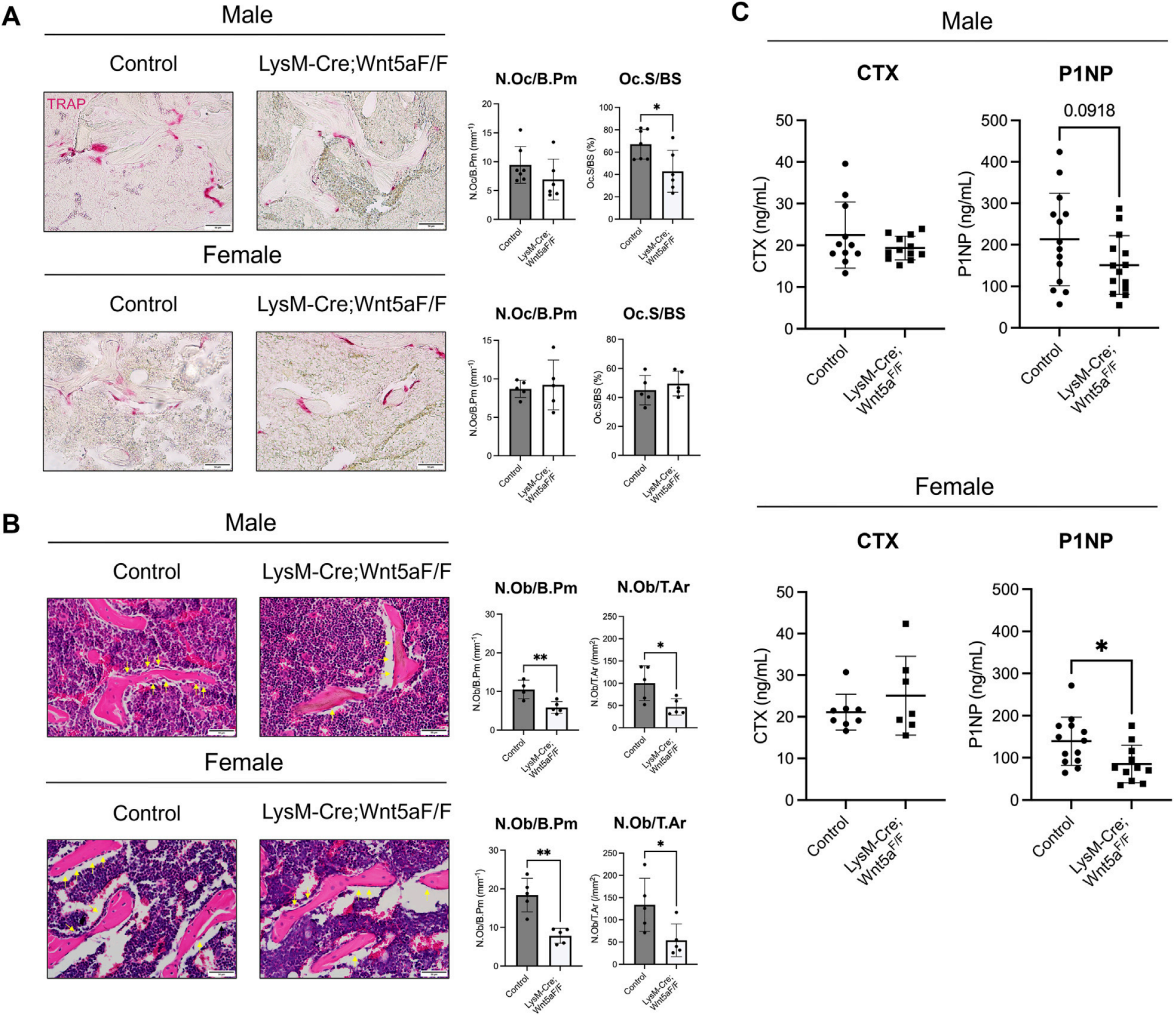
Conditional deletion of *Wnt5a* in osteoclast precursors affects osteoblasts, but not osteoclasts, *in vivo*. **(A,B)** The number of TRAP + osteoclasts and osteoblasts, based on their morphology on H&E-stained sections, was quantified by histomorphometry on distal femur sections. **(A)** The number of osteoclasts (N.Oc/B.Pm) is unaffected by the mutation, while osteoclast surface (Oc.S/BS) is significantly reduced in male *Wnt5a* cKO. **(B)** Osteoblast numbers (N.Ob/B.Pm and N. Ob/T.Ar) are significantly decreased in male and female *Wnt5a* cKO compared to controls (males n = 4–7/genotype; females n = 5/genotype). Statistical analysis was performed by unpaired, two-tailed *t*-test. *p < 0.05, **p < 0.01. **(C)** Serum levels of CTX were unaffected by the mutation. On the contrary, P1NP serum levels were significantly reduced in female, with a trend towards a reduction in male, *Wnt5a* cKO compared to controls (males n = 11–14/genotype; females n = 7–13/genotype). Statistical analysis was performed by unpaired, two-tailed *t*-test. *p < 0.05. N. Oc/B.Pm, osteoclast number over bone perimeter; Oc. S/BS, osteoclast surface; N. Ob/B.Pm, osteoblast number over bone perimeter; N. Ob/T.Ar, osteoblast number over tissue area; P1NP, N-terminal propeptide of type I procollagen; CTX, C-terminal telopeptide of collagen type I.

Bone formation and bone resorption are tightly coupled processes. To investigate whether changes in bone resorption *in vivo* contributed to the observed osteopenic phenotype, osteoclast and osteoblast number and surface were evaluated in histological femur sections. No significant changes in osteoclast number over bone perimeter (N.Oc/B.Pm) or osteoclast surface over bone surface (Oc.S/BS) were detected between female *Wnt5a* cKO and control mice (Figure 4A). Male *Wnt5a* cKO mice did not differ in osteoclast number compared to their controls, but osteoclast surface was reduced, consistent with the *in vitro* data (Figure 4A). In contrast, osteoblast numbers, measured over bone perimeter (N.Ob/B.Pm) and tissue area (N.Ob/T.Ar) were significantly reduced in both male and female *Wnt5a* cKO mice compared to controls (Figure 4B). To quantify bone formation and bone resorption *in vivo*, we analyzed serum levels of N-terminal propeptide of type I procollagen (P1NP) and C-terminal collagen cross-links (CTX), respectively. Interestingly, *Wnt5a* cKO presented reduced levels of P1NP compared to controls, although only females reached statistical significance (Figure 4C), indicating that low bone mass in *Wnt5a* cKO mice may be caused by decreased bone formation. Furthermore, conditional deletion of *Wnt5a* in myeloid cells did not influence serum levels of the bone resorption biomarker CTX in either male or female mice (Figure 4C), supporting the conclusion that osteoclast activity *in vivo* is not affected by loss of osteoclast-derived *Wnt5a*.

### 3.5 Functional analysis identifies dysregulation of ECM dynamics and enhanced inflammatory signaling in *Wnt5a* cKO

**FIGURE 5:**
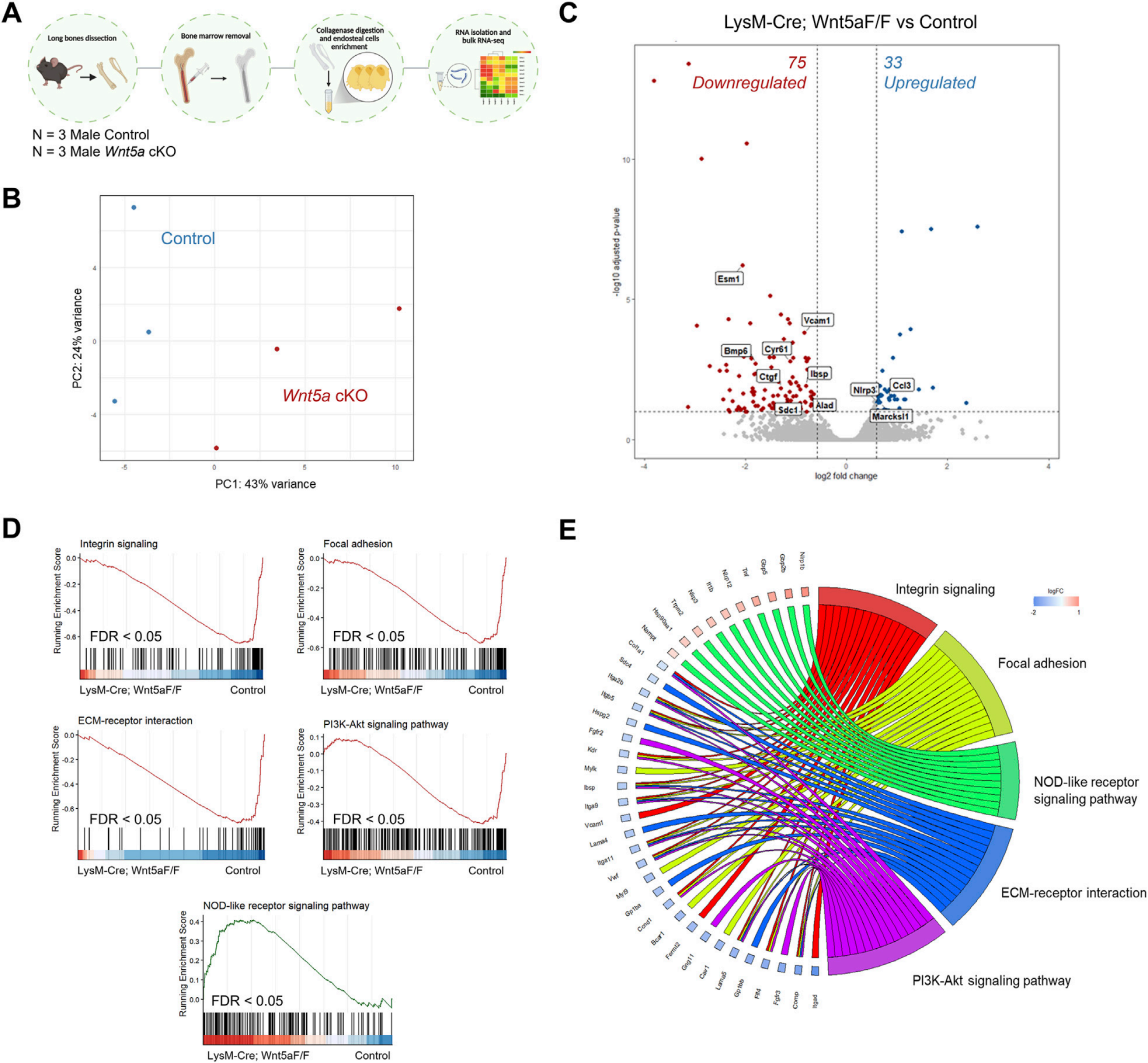
Functional analysis of endosteal cells from *Wnt5a* cKO mice compared to controls. **(A)** Experimental design for endosteal cell isolation and bulk RNA-Seq. **(B)** Principal component analysis (PCA) to visualize sample to sample variation. The PCA plot depicted clustering control versus *Wnt5a* cKO samples. PC1 and PC2 components explained 43% and 24% of the variability in the expression data, respectively. **(C)** Volcano plots showing the differentially expressed genes (DEGs) in *Wnt5a* cKO compared to controls (cutoff: |FC| > 1.5 and FDR <0.05). DEGs known to interact with or act downstream of WNT5A, according to the literature, were annotated on the graph. **(D)** Gene set enrichment analysis (GSEA) on the entire transcriptome profile demonstrated downregulation of the Integrin signaling, Focal adhesion, ECM-receptor interaction, and PI3K-Akt signaling pathway, and upregulation of NOD-like receptor signaling pathway in *Wnt5a* cKO endosteal cells. **(E)** The leading-edge genes associated with these pathways were shown using a chord diagram.

To investigate the effect of osteoclast-derived *Wnt5a* on osteoblast function, we performed bulk RNA-seq analysis of osteoblast-enriched endosteal cells from male *Wnt5a* cKO mice and respective controls (Figure 5A). On average, we obtained 37 million reads/library, with >80% of reads uniquely aligning to the annotated genome.

The transcriptional profile showed abundant expression of osteoblastic and mesenchymal genes (*Runx2, Bglap, Alpl, Col1a1, Col1a2, Bmp6, Comp, Ibsp, Spp1*), consistent with an osteoblast-lineage signature. However, transcripts associated with additional cell types were also detected. Similar findings have been reported in studies using the same enzymatic digestion protocol to isolate cells from the endosteal surface, indicating that this approach yields a heterogeneous cell population rather than a pure osteoblast fraction (Ayturk et al., 2020; Ayturk et al., 2013). Accordingly, while enriched for osteoblast-lineage cells, our preparation represents a mixed population and is therefore referred to here as endosteal cells.

Principal component analysis (PCA) revealed separation between Control and *Wnt5a* cKO samples, with 43% variance along PC1. Variability along PC2 (24%) reflected intra-group heterogeneity, with comparable within-group dispersion (Figure 5B). Differential expression analysis revealed 75 downregulated and 33 upregulated genes in endosteal cells from *Wnt5a* cKO compared to controls (Figure 5C; Supplementary Table S2). Notably, several differentially expressed genes (DEGs) have been reported to interact with or be expressed downstream of Wnt5a in skeletal and non-skeletal contexts (Figure 5C). Among these, *Cyr61* (*Ccn1*) and *Ctgf* (*Ccn2*) are well-established downstream effectors of Wnt5a-dependent signaling, playing critical roles in coupling extracellular matrix cues to cellular responses, including osteoblast differentiation and function (Park et al., 2015; Tarr et al., 2018; Hendesi et al., 2015).

To investigate the biological processes affected by loss of osteoclast-derived WNT5A, we performed gene ontology (GO) analysis on the DEGs identified in endosteal cells from *Wnt5a* cKO mice compared to controls. Enriched biological processes included regulation of cell communication, differentiation, and signal transduction, but also immune system process and defense response (Supplementary Figure S4A). Molecular function analysis showed an enrichment of genes involved in protein binding, signaling receptor binding, glycosaminoglycan binding, and integrin binding (Supplementary Figure S4B). Consistent with these findings, cellular component analysis showed enrichment of extracellular and cell-surface terms, including extracellular region, extracellular matrix, collagen-containing extracellular matrix, collagen trimer, cell surface, and vesicle-associated terms (Supplementary Figure S4C).

Pathway analysis further revealed downregulation of adhesion- and matrix-related pathways, including Integrin signaling, ECM-receptor interaction, Focal adhesion, and PI3K-Akt signaling pathways (Figures 5D,E). In contrast, NOD-like receptor signaling emerged as the main significantly upregulated pathway in *Wnt5a* cKO mice. Together, these findings support a model in which osteoclast-derived WNT5A shapes the extracellular signaling environment by regulating integrin-mediated cell adhesion to the matrix.

### 3.6 Extracellular matrix maturation is negatively affected in *Wnt5a* cKO long bones

**FIGURE 6:**
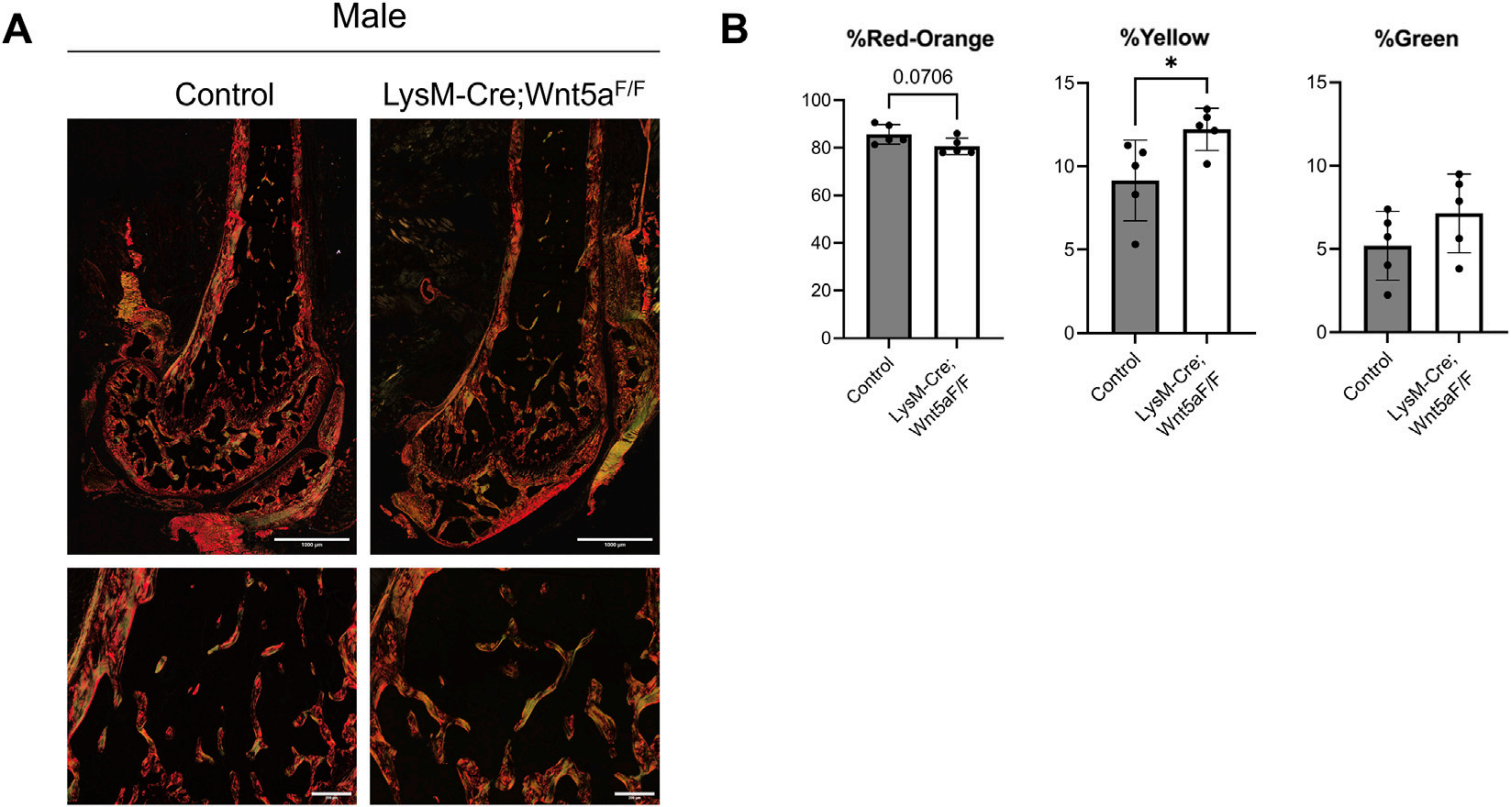
Conditional deletion of Wnt5a in osteoclast precursors affects ECM quality. **(A,B)** Picro Sirius Red staining of collagen fibers in femur sections revealed significant increase in the percentage of less mature yellow fibers, with a trend towards a reduction in the percentage of mature, red-orange fibers in male Wnt5a cKO compared to controls, indicating decreased collagen maturation (n = 5/genotype). Statistical analysis was performed by unpaired, two-tailed t-test. * p < 0.05.

To assess if the composition of the ECM was changed in response to *Wnt5a* deletion in osteoclasts *in vivo*, Picro Sirius Red staining was performed on femur sections, and the ratio of mature to immature collagen was analyzed under polarized light, as previously described (Vennin et al., 2019). We observed a trend toward decreased mature collagen (% red-orange fibers) in both female and male *Wnt5a* cKO (Figure 6; Supplementary Figure S5), while the proportion of immature collagen (% green fibers) remained unchanged. Notably, male *Wnt5a* cKO displayed a significant increase in less mature collagen (% yellow fibers), indicating altered collagen maturation (Figure 6). These findings align with our transcriptomic data and support a model in which loss of osteoclast-derived Wnt5a leads to subtle but functionally relevant alterations in ECM quality and organization, rather than gross changes in collagen content.

### 3.7 Loss of osteoclast-derived *Wnt5a* disrupts focal adhesions in MC3T3 pre-osteoblasts

**FIGURE 7:**
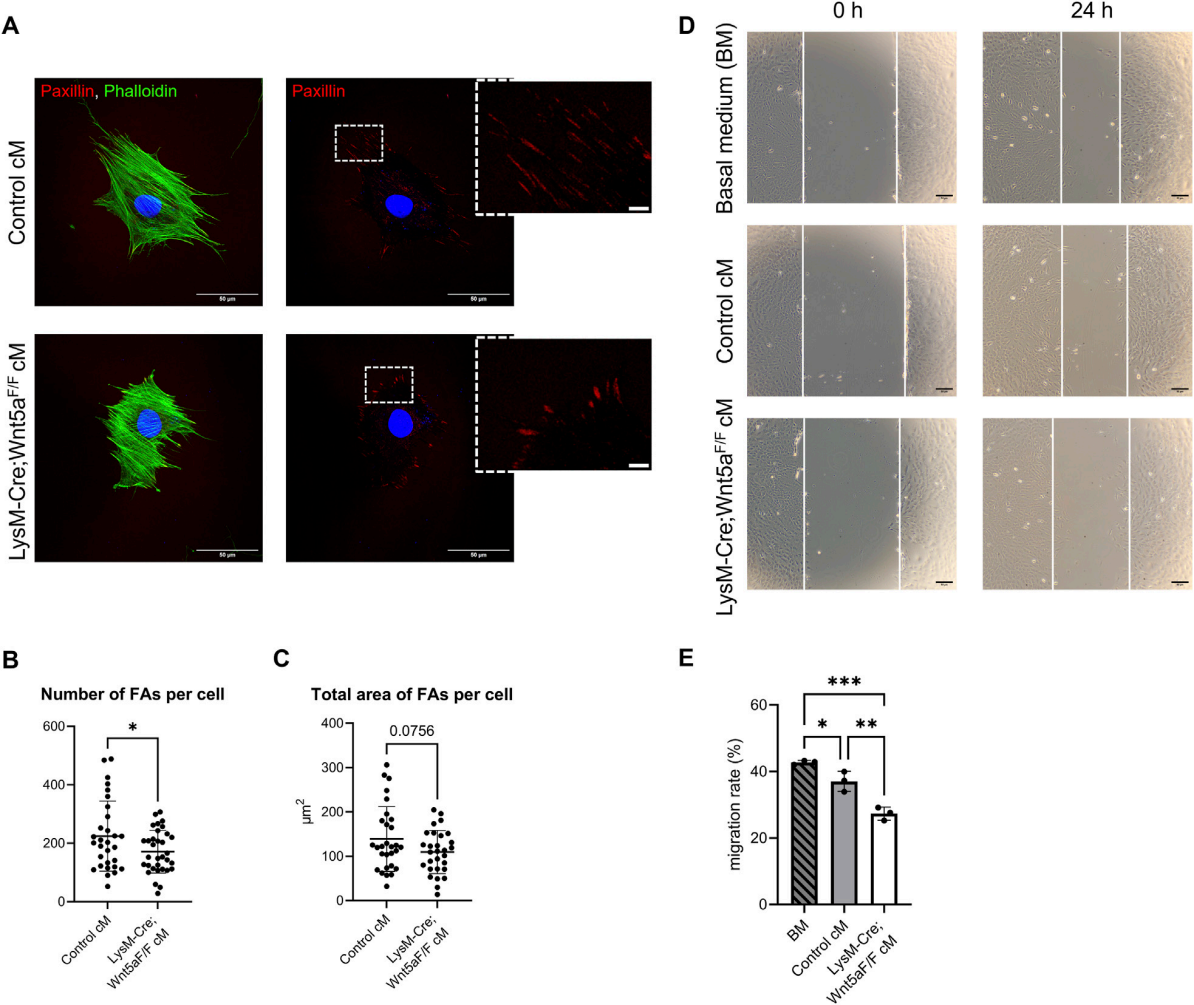
Treatment of MC3T3 preosteoblasts with conditioned medium from *Wnt5a* cKO osteoclasts results in decreased focal adhesions and migration rate. **(A)** Confocal images of 24-h control and *Wnt5a* cKO cM-treated MC3T3 stained with anti-Paxillin (red) and Phalloidin (anti-F-actin, green). Scale bar inserts = 5 µm. **(B)** The number of focal adhesion per cell was significantly decreased in the *Wnt5a* cKO cM-treated group. **(C)** The total area of focal adhesion per cell showed a trend towards decreased area in the *Wnt5a* cKO cM-treated group but did not reach significance (biological replicates n = 3/group, n = 29–33 cells). Statistical analysis was performed by unpaired, two-tailed *t*-test. *p < 0.05. **(D)** Wound-healing assay. Cell migration of MC3T3 cells in basal medium (BM), and treated with control and *Wnt5a* cKO cM was assessed after 24h and compared with the area of the initial scratch (0h) (biological replicates n = 3/group). **(E)** Migration rate was significantly decreased in *Wnt5a* cKO cM-treated cells compared to the other two groups. Statistical analysis was performed by one-way ANOVA followed by Tukey’s *post hoc* test. *p < 0.05, **p < 0.01, ***p < 0.001.

Our transcriptomic analyses suggested that impaired adhesion may contribute to the low bone mass phenotype observed in *Wnt5a* cKO mice. To functionally test this hypothesis, we assessed focal adhesion formation in MC3T3 pre-osteoblasts exposed to conditioned medium (cM) derived from control or *Wnt5a* cKO osteoclasts through Paxillin staining (Figure 7A). We selected Paxillin as a focal adhesion readout, due to its role as a core adaptor protein of integrin-based adhesion complexes and a well-established downstream effector of non-canonical WNT5A signaling (Wei et al., 2013). Consistent with our transcriptomic results, MC3T3 cells cultured in cM from *Wnt5a* cKO osteoclasts exhibited a significant reduction in the number of focal adhesions per cell (Figure 7B), together with a trend toward reduced total focal adhesion area per cell (Figure 7C).

Focal adhesions can regulate a variety of cellular processes, including cell migration (Zebda et al., 2012). To investigate whether the impairment of focal adhesion dynamics observed upon loss of osteoclast-derived WNT5A would translate into altered migratory behavior, we performed a wound healing assay on MC3T3 cells treated for 24 h with cM from control or *Wnt5a* cKO osteoclasts (Figure 7D). A basal medium (BM) group was also included to define baseline motility. While both control and *Wnt5a* cKO cM-treated cells displayed a reduced migration rate compared to the BM group, MC3T3 cells exposed to *Wnt5a* cKO cM showed a further significant reduction in migration rate compared to cells treated with control cM (Figure 7E). These results demonstrate that loss of osteoclast-derived Wnt5a is associated with impaired osteoblast migration, consistent with the defects in focal adhesion number and organization.

## 4 Discussion

Current therapies for osteoporosis remain limited due to off-target effects resulting, in some settings, in adverse events (Kennel and Drake, 2009). Antiresorptive agents such as bisphosphonates are highly effective, yet long-term use has been associated with rare but serious complications, including atypical femoral fractures and osteonecrosis of the jaw (Kennel and Drake, 2009). These limitations underscore the need for strategies that enhance bone formation in a more physiological manner by preserving the coupling between osteoclasts and osteoblasts during bone remodeling. Within this context, our findings identify osteoclast-derived WNT5A as a candidate coupling factor that supports bone formation without measurably increasing osteoclast number or resorptive activity.

Using *Ctsk-Cre*, we previously showed that conditional deletion of *Wnt5a* in mature osteoclasts led to a decrease in trabecular bone in male mice (Roberts et al., 2020). However, due to the expression of *Ctsk* in periosteal and other mesenchymal cells (Debnath et al., 2018; Zou et al., 2022), we could not exclude that deletion of *Wnt5a* from these cells contributed to the observed phenotype. Furthermore, inhibition of *Ctsk* expression by estrogen likely affected Cre-mediated WNT5A transgene recombination efficiency in females, restricting our analyses to male mice (Roberts et al., 2020; Troen, 2006). In the present study, to exclude that deletion of *Wnt5a* from mesenchymal cells contributes to the observed skeletal phenotypes, we used LysM-Cre to target *Wnt5a* in myeloid lineage cells, including osteoclast precursor cells, thereby also targeting their progeny, mature osteoclasts. This strategy enabled us to interrogate a broader portion of the osteoclast lineage, providing a more comprehensive investigation of osteoclast-derived WNT5A effects while leaving mesenchymal cell-produced WNT5A intact. We found that the skeletal phenotype observed in *LysM-Cre;Wnt5a* ^*F/F*^ mice is consistent with our previous *Ctsk-Cre; Wnt5a* ^*F/F*^ model. The convergence of both models on a similar low bone mass phenotype driven by impaired osteoblast function, without changes in osteoclast activity, strongly suggests that the effect is osteoclast-lineage dependent with minimal contribution from mesenchymal or other myeloid cells. Importantly, *LysM-Cre* does not show sex-dependent effects on recombination efficiency, allowing the analyses of both male and female mice.

We detected significantly reduced body weight during development in female, but not male, *Wnt5a* cKO mice. Previous work demonstrated that macrophage-derived *Wnt5a* affects glucose metabolism and inflammation in white adipose tissue following high-fat diet (Fuster et al., 2015). However, this study did not report any differences in body weight between *LysM-Cre; Wnt5a* ^*F/F*^ and control mice nor did the authors specify the sex of the mice analyzed. Our data raise the possibility that myeloid-derived WNT5A contributes to metabolic regulation in a sex-specific manner, consistent with the existing literature. Further studies will be required to clarify the mechanisms underlying the observed differences in body weight between male and female *Wnt5a* cKO mice.

In this work, the low bone mass phenotype in both male and female *Wnt5a* cKO mice strengthens the conclusion that osteoclast-lineage WNT5A contributes meaningfully to skeletal homeostasis. We show that this is primarily due to impaired osteoblast function. This is supported by decreased serum levels of the bone formation marker P1NP, which reached statistical significance in females and showed a similar trend in males. Moreover, our multifaceted evaluations of osteoclast formation and activity revealed that the phenotype is unlikely due to a change in bone resorption. Although we did not directly perform calcein double-labeling to assess dynamic bone formation by histomorphometry, which is a limitation of our study, the clear dissociation between bone resorption and bone mass supports a model in which osteoclast-derived WNT5A functions as a paracrine coupling factor that promotes osteoblast activity and bone formation.

Our transcriptomic analyses help explain the phenotype by pointing to a convergent defect in osteoblast-matrix interactions. WNT5A has been extensively shown to regulate cell migration, polarity, and adhesion through non-canonical pathways converging on cytoskeletal remodeling and integrin-dependent signaling (Dejmek et al., 2003; Kawasaki et al., 2007; Matsumoto et al., 2010; Zhang et al., 2014). Consistent with this body of evidence, multiple downregulated genes in our dataset encode matricellular or ECM-associated proteins that directly engage integrins and modulate focal adhesion signaling, including *Cyr61* (*Ccn1*), *Ctgf* (*Ccn2*), *Ibsp*, *Sdc1*, *Vcam1*, *Marcksl1*, *Bmp6* and *Esm1*, all previously reported to function downstream of WNT5A-dependent signaling in skeletal and non-skeletal contexts (Park et al., 2015; Tarr et al., 2018; Peng et al., 2010; O'Connell et al., 2009; Herr et al., 2014; Mohapatra et al., 2020; Lee et al., 2014; Xu et al., 2025). These genes collectively support a biologically coherent model in which loss of osteoclast-derived WNT5A weakens the adhesive and migratory programs required for efficient osteoblast recruitment and function on bone surfaces.

GSEA-KEGG further revealed downregulation of ECM-receptor interaction, focal adhesion, integrin signaling, and PI3K-Akt signaling pathways. These pathways represent sequential biological layers of cell-matrix communication, from extracellular ligand availability to intracellular adhesion complex assembly and downstream signaling. The coordinated suppression of these pathways provides a coherent mechanistic framework linking loss of osteoclast-derived WNT5A to reduced osteoblast number and anabolic output, despite preserved osteoclast activity. Interestingly, GSEA-KEGG revealed upregulation of the stress-related NOD-like receptor signaling pathway, driven by genes including *Tnf*, *Nlrp3*, and *Il1b*. While this signature is classically associated with pro-osteoclastogenic inflammation (Ke et al., 2017; Kwon et al., 2021), in *Wnt5a* cKO mice it did not translate into a strong resorptive phenotype. While we cannot exclude macrophage activation due to global targeting of the myeloid lineage by *LysM-Cre*, we propose that this represents a secondary, stress-associated response to disrupted integrin signaling and altered cell–matrix interactions, consistent with emerging evidence linking ECM and integrin signaling to inflammasome activation (Thinwa et al., 2014; Frevert et al., 2018; Mezu-Ndubuisi and Maheshwari, 2021).

Our *in vitro* conditioned medium experiments provide functional evidence that loss of osteoclast-derived WNT5A directly impairs osteoblast focal adhesion dynamics and migratory behavior. MC3T3 cells exposed to conditioned medium from WNT5A-deficient osteoclasts exhibited fewer focal adhesions, despite being seeded on a uniform collagen type I-coated substrate. This experimental design isolates osteoblast-intrinsic adhesion defects from matrix availability, indicating that impaired focal adhesion assembly occurs upstream of extracellular matrix alterations. The concomitant delay in cell migration links focal adhesion instability to reduced osteoblast motility and offers a plausible cellular explanation for the reduced osteoblast number observed in trabecular bone. These findings align with prior reports demonstrating that WNT5A regulates focal adhesion turnover, cytoskeletal organization, and cell migration through non-canonical signaling pathways involving integrin engagement and PI3K-Akt signaling (Dejmek et al., 2003; Kawasaki et al., 2007; Matsumoto et al., 2010; Zhang et al., 2014). More broadly, since focal adhesion is a critical determinant of osteoblast differentiation, survival, and mechanosensitive anabolic responses (Sun et al., 2016; Zhao et al., 2021), our data support a model in which osteoclast-derived WNT5A sustains osteoblast recruitment and function by maintaining adhesion-dependent signaling. However, it should be noted that conditioned medium experiments cannot establish that the observed effects on osteoblasts are directly mediated by WNT5A itself, as broader changes in the secretome of WNT5A-deficient osteoclasts may also contribute. Future experiments employing rescue with purified recombinant WNT5A protein or WNT5A-neutralizing antibodies will be required to establish a more direct causal relationship. Additionally, osteoclast numbers were not normalized, and total protein normalization of the conditioned medium was not performed, which represents a methodological limitation of our assay.

Picro Sirius Red staining revealed modest alterations in collagen maturation in male *Wnt5a* cKO mice, with a shift toward less mature collagen fibers, consistent with subtle disruption of ECM quality. These changes were not observed in females. We hypothesize that this sex-specific impairment in collagen maturation is the primary driver of the biomechanical deficits observed exclusively in male *Wnt5a* cKO mice, as bone toughness and resistance to torsional failure are highly dependent on collagen fiber organization and maturation (Unal et al., 2018). In females, the preservation of collagen maturation may reflect estrogen-mediated compensation, as estrogen is known to promote collagen crosslinking through upregulation of lysyl oxidase activity in bone (Viguet-Carrin et al., 2006; Sanada et al., 1978), potentially masking the consequences of reduced trabecular volume on bone strength. Additionally, males typically have higher collagen production and a larger total collagen content (Tarnutzer et al., 2023), which may exacerbate the functional consequences of impaired matrix quality. Picro Sirius birefringence analysis, however, may not fully capture ECM architectural changes at the fibrillar level. Transcriptomic downregulation of *Col5a3* and *Col14a1* suggests that the primary disruption may involve collagen fibrillogenesis and crosslinking rather than overall collagen maturation, an area warranting further investigation. Together with our biomechanical findings, these data reinforce the view that osteoclast-derived WNT5A is required not only for osteoblast recruitment but also for the quality of the bone matrix they produce, a distinction with direct relevance to bone fragility and fracture risk.

**FIGURE 8:**
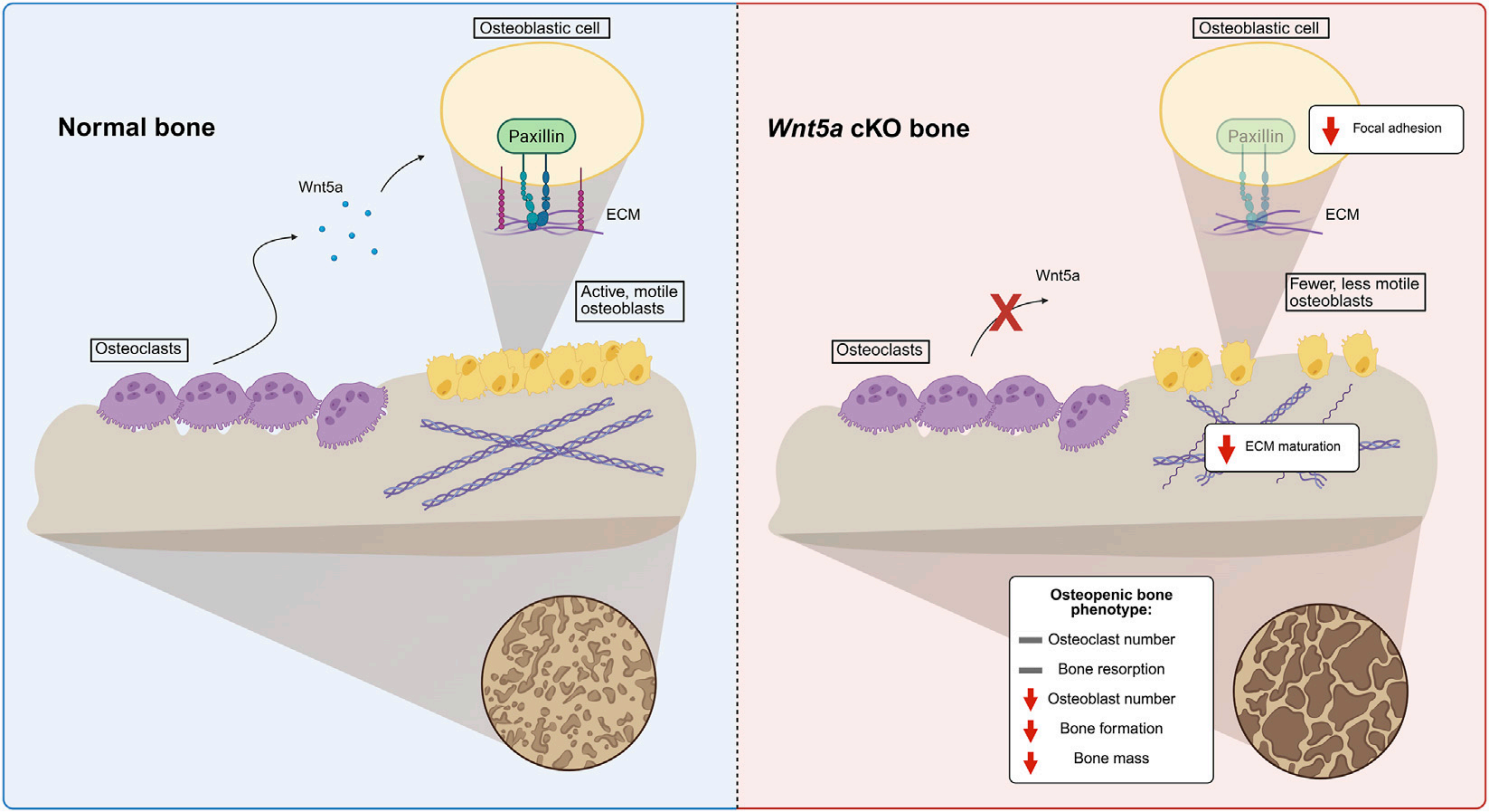
Schematic model of osteoclast-derived WNT5A as an anabolic clastokine regulating osteoblast adhesion, migration and bone formation. In normal bone (left), osteoclasts secrete WNT5A, which acts in a paracrine manner on osteoblastic cells to promote focal adhesion via paxillin, supporting integrin-ECM interactions, and maintaining osteoblast motility and matrix production, collectively sustaining bone formation. In *Wnt5a* cKO bone (right), loss of osteoclast-derived WNT5A impairs focal adhesion assembly and reducing osteoblast motility, resulting in fewer, less active osteoblasts and decreased bone formation without changes in osteoclast number or resorptive activity. Ultimately, this leads to an osteopenic bone phenotype. Created with BioRender.com.

In conclusion, we propose that osteoclast-derived WNT5A acts as a paracrine coupling signal that maintains an adhesion-competent osteoblast state within the bone microenvironment (Figure 8). Its loss disrupts focal adhesion signaling, impairing osteoblast migration and matrix production, resulting in reduced bone mass without changes in osteoclast number or activity. These findings strengthen emerging concepts highlighting the instructive role of osteoclasts in osteoblast recruitment, differentiation, and function through secretion of clastokines (Sims and Martin, 2014; Daponte et al., 2024), and provide a mechanistic foundation for therapeutic strategies targeting osteoclast-osteoblast coupling. The difference between the identified function of osteoblast- and osteoclast-derived *Wnt5a* underscores the importance of further research into the cell-of-origin–specific mechanisms of *Wnt5a* action to facilitate the development of a *Wnt5a*-based anabolic treatment strategy.

## Data Availability

The datasets presented in this study can be found in online repositories. The names of the repository/repositories and accession number(s) can be found below: https://www.ncbi.nlm.nih.gov/geo/, GSE324013.
