# Supplementary material for: Conditional deletion of Wnt5a in myeloid osteoclast precursors results in low bone mass via disruption of osteoblast migration and focal adhesion dynamics
Source: Front Cell Dev Biol. 2026 Jul 22;14:1868995. doi: 10.3389/fcell.2026.1868995 (PMC13437647; doi:10.3389/fcell.2026.1868995)
Supplement: Supplementary file 1 [file DataSheet1.docx]

Supplementary Material

# 1 Supplementary Figures and Tables

Supplementary Table 1. Primers used for qPCR on BMMs.

| **Gene** | **Primer sequences (5’-3’)** | **Amplicon size** | **Ensembl ID** |
| --- | --- | --- | --- |
| *Acp5 (TRAP)* | CCAATGCCAAAGAGATCGCC  TCTGTGCAGAGACGTTGCCAA | 216 bp | ENSMUSG00000001348 |
| *Calcr* | GAGGTTCCTTCTCGTGAACAG  AGTCAGTGAGATTGGTAGGAGC | 92 bp | ENSMUSG00000023964 |
| *Ctsk* | CTCGGCGTTTAATTTGGGAGA  TCGAGAGGGAGGTATTCTGAGT | 164 bp | ENSMUSG00000028111 |
| *Wnt5a* | TCTCCTTCGCCCAGGTTGTTATAG  TCCTGATACAAGTGGCAGAGTT | 163 bp | ENSMUSG00000021994 |
| *β-actin* | AGATGTGGATCAGCAAGCAG  GCGCAAGTTAGGTTTTGTCA | 125 bp | ENSMUSG00000029580 |

Supplementary Table 2. Complete list of differentially expressed genes (DEGs) identified by RNA-Seq.

| **Gene** | **ensembl_ID** | **log2FC** | **adjPval** | **Control1** | **Control2** | **Control3** | **Wnt5acKO1** | **Wnt5acKO2** | **Wnt5acKO3** |
| --- | --- | --- | --- | --- | --- | --- | --- | --- | --- |
| Ighv1-76 | ENSMUSG00000093896 | -3.133857915 | 4.10E-14 | 9.940232 | 8.707414 | 9.468445 | 6.431309737 | 6.398735428 | 6.324348382 |
| Igkv16-104 | ENSMUSG00000076522 | -3.815690549 | 1.66E-13 | 8.95602 | 10.11175 | 8.231919 | 5.43106405 | 5.831106038 | 5.532841991 |
| Igkv10-96 | ENSMUSG00000094420 | -1.979681449 | 2.77E-11 | 10.29332 | 11.0641 | 11.07859 | 8.996770974 | 9.018458762 | 8.605002586 |
| Ighv14-2 | ENSMUSG00000095583 | -2.873062323 | 9.93E-11 | 8.978919 | 9.904493 | 10.65539 | 7.17506893 | 7.48745169 | 6.734064111 |
| GM29376 | GM29376 | 2.582420392 | 2.57E-08 | 6.69888 | 6.548336 | 6.707253 | 9.065947002 | 9.754244922 | 8.446894188 |
| GM8210 | GM8210 | 1.669241212 | 3.07E-08 | 9.569794 | 9.447318 | 9.395805 | 10.97754196 | 11.61500691 | 10.64385706 |
| BC018473 | BC018473 | 1.0936919 | 3.82E-08 | 14.33037 | 14.62478 | 14.09875 | 15.33561182 | 15.60745794 | 15.42648675 |
| Esm1 | ENSMUSG00000042379 | -2.057670741 | 6.05E-07 | 9.4296 | 9.640391 | 9.748389 | 7.612512611 | 8.032247219 | 6.856134882 |
| Stab2 | ENSMUSG00000035459 | -1.515245944 | 7.33E-06 | 10.86098 | 11.38879 | 10.73198 | 9.180179764 | 9.261319516 | 9.96218157 |
| Acp1 | ENSMUSG00000044573 | -1.309353318 | 3.37E-05 | 10.67818 | 10.47316 | 11.24692 | 9.483019268 | 9.647213947 | 9.461863511 |
| Axl | ENSMUSG00000002602 | -1.168882682 | 5.15E-05 | 11.78806 | 11.82695 | 12.56639 | 10.86791664 | 10.99976689 | 10.94903356 |
| Igkv17-127 | ENSMUSG00000076508 | -2.338526915 | 5.15E-05 | 8.466001 | 10.31126 | 9.22229 | 7.733111439 | 6.878977078 | 6.890829055 |
| C1qa | ENSMUSG00000036887 | -1.125874719 | 6.94E-05 | 10.98736 | 10.89149 | 11.47658 | 10.04006945 | 10.09534013 | 9.916608334 |
| Fcna | ENSMUSG00000026938 | -1.909647643 | 7.16E-05 | 10.9572 | 9.962919 | 11.56803 | 9.302173252 | 8.171183502 | 9.434688032 |
| Ighv1-50 | ENSMUSG00000094198 | -2.968821019 | 8.53E-05 | 8.590172 | 8.071505 | 9.811805 | 7.00512559 | 5.806050309 | 4.625180335 |
| Aph1b | ENSMUSG00000032375 | 1.257989511 | 0.000112426 | 9.457818 | 9.827219 | 9.62494 | 11.2964529 | 10.79613732 | 10.48640598 |
| Vcam1 | ENSMUSG00000027962 | -0.843547196 | 0.000154768 | 13.35171 | 13.54109 | 13.86368 | 12.80173292 | 12.76371767 | 12.70712217 |
| GYK | ENSMUSG00000025059 | 1.054507441 | 0.000173277 | 9.427223 | 9.284499 | 9.601803 | 10.47999573 | 10.57851937 | 10.41867299 |
| Fabp4 | ENSMUSG00000062515 | -1.246300552 | 0.00025482 | 11.7022 | 12.10104 | 12.01226 | 11.24666927 | 10.46705009 | 10.18005059 |
| C1qb | ENSMUSG00000036905 | -1.070725414 | 0.000349169 | 11.18388 | 10.90928 | 11.59126 | 10.29989456 | 10.23565173 | 10.01162326 |
| Igkv17-121 | ENSMUSG00000076514 | -2.03290594 | 0.001117867 | 8.874629 | 9.431641 | 10.98195 | 8.097970391 | 8.372338931 | 7.504991751 |
| Treml1 | ENSMUSG00000023993 | -1.441346859 | 0.001125738 | 9.61435 | 10.24104 | 10.79453 | 8.670574516 | 8.633180483 | 9.208127493 |
| Angptl4 | ENSMUSG00000002289 | -1.528083855 | 0.001125738 | 9.776238 | 10.30159 | 9.830298 | 8.927088011 | 8.563873604 | 7.647065132 |
| Oasl1 | ENSMUSG00000041827 | 0.915336033 | 0.001197168 | 10.09048 | 10.22976 | 10.24872 | 11.29010626 | 10.82993519 | 11.15573541 |
| Lyz1 | ENSMUSG00000069515 | -0.813657945 | 0.001228481 | 13.64384 | 13.76909 | 13.47983 | 12.96474662 | 12.9797321 | 12.4662341 |
| Fcgrt | ENSMUSG00000003420 | -1.056104561 | 0.001228481 | 10.81173 | 11.11283 | 11.09397 | 10.05647966 | 10.22505731 | 9.503559754 |
| Pxk | ENSMUSG00000033885 | -0.763884223 | 0.001256488 | 11.65677 | 11.70102 | 11.78661 | 10.98613378 | 11.05198566 | 10.81093011 |
| Chrdl1 | ENSMUSG00000031283 | -1.884316918 | 0.001286261 | 8.191365 | 9.335934 | 8.308667 | 6.923979279 | 6.779510824 | 6.879356785 |
| Lyz2 | ENSMUSG00000069516 | -0.794882357 | 0.001630281 | 16.67294 | 16.71983 | 16.51397 | 16.0117698 | 16.01851684 | 15.4228796 |
| CYR61 | ENSMUSG00000028195 | -1.116690214 | 0.001630281 | 11.05665 | 11.17064 | 10.35046 | 9.996791724 | 9.729472222 | 9.61324043 |
| Bmp6 | ENSMUSG00000039004 | -1.801905237 | 0.001934127 | 7.927797 | 8.992926 | 8.685683 | 6.863089511 | 6.994379531 | 6.628378751 |
| Ighv1-84 | ENSMUSG00000094940 | -2.387360119 | 0.002172953 | 8.17446 | 7.117505 | 8.11611 | 6.113508912 | 5.366207747 | 5.090758466 |
| Col14a1 | ENSMUSG00000022371 | -2.709767456 | 0.002398339 | 6.459372 | 8.467141 | 8.591251 | 5.786266223 | 5.855734035 | 4.569158566 |
| Tgfb1i1 | ENSMUSG00000030782 | -1.495042304 | 0.002592259 | 8.652541 | 9.291029 | 9.245443 | 7.308937676 | 7.954569043 | 7.504991751 |
| Ncoa4 | ENSMUSG00000056234 | -0.785712335 | 0.003192269 | 11.97499 | 11.79399 | 12.15175 | 10.97466661 | 11.34619762 | 11.24280467 |
| Apoe | ENSMUSG00000002985 | -0.674683962 | 0.003275627 | 14.306 | 14.55764 | 14.55257 | 13.80072006 | 13.97340898 | 13.60979142 |
| Csf3r | ENSMUSG00000028859 | 0.707313556 | 0.003478715 | 14.19539 | 14.24023 | 13.82213 | 14.65094824 | 14.77323075 | 14.97209143 |
| Ighv1-59 | ENSMUSG00000095197 | -2.378427511 | 0.003505617 | 7.941185 | 7.859395 | 10.08382 | 7.271939996 | 6.364820376 | 6.200747833 |
| Ighv1-9 | ENSMUSG00000094694 | -2.512311337 | 0.003517432 | 7.801363 | 9.284499 | 9.499592 | 7.641809096 | 5.433195873 | 5.410650652 |
| Heph | ENSMUSG00000031209 | -1.808581138 | 0.004366146 | 8.581648 | 8.330114 | 8.06497 | 7.071222171 | 6.201488199 | 6.290101112 |
| Itgad | ENSMUSG00000070369 | -1.82286128 | 0.004526333 | 9.12392 | 8.992926 | 10.41825 | 7.406045067 | 7.266478164 | 8.544548948 |
| Igkv4-57 | ENSMUSG00000076556 | -2.130512263 | 0.005231386 | 7.435694 | 8.623658 | 9.17725 | 7.049524711 | 5.754594356 | 6.406570196 |
| C4b | ENSMUSG00000073418 | -1.072549408 | 0.00584173 | 10.38924 | 11.05517 | 11.15393 | 10.15120413 | 9.800965227 | 9.473355249 |
| Ighv1-26 | ENSMUSG00000094546 | -1.510226412 | 0.007354868 | 9.483209 | 9.64889 | 10.20923 | 8.103245616 | 8.974156745 | 7.482420454 |
| Mrc1 | ENSMUSG00000026712 | -1.125448501 | 0.008739381 | 10.91457 | 11.43178 | 12.01832 | 9.872546752 | 10.64449479 | 10.57084888 |
| Mertk | ENSMUSG00000014361 | -1.347825663 | 0.008849396 | 9.791101 | 8.92457 | 9.616085 | 7.746658903 | 8.085277678 | 8.522592795 |
| Pf4 | ENSMUSG00000029373 | -1.001052245 | 0.00924879 | 11.72125 | 12.11521 | 12.70896 | 11.3096336 | 11.50058428 | 10.82967879 |
| Mafb | ENSMUSG00000074622 | -1.106553182 | 0.009761995 | 9.341472 | 9.700495 | 9.511101 | 8.681196013 | 8.359479871 | 8.201821727 |
| Slfn4 | ENSMUSG00000000204 | 0.653844886 | 0.011828805 | 14.2564 | 14.291 | 14.16794 | 15.12386617 | 14.57833363 | 14.92531056 |
| Ibsp | ENSMUSG00000029306 | -0.800856799 | 0.012319247 | 11.86207 | 12.0244 | 12.24688 | 11.58836651 | 10.98449338 | 11.11535248 |
| MRVI1 | ENSMUSG00000005611 | -0.954672102 | 0.012319247 | 10.05089 | 10.4314 | 10.61862 | 9.605176249 | 9.168529747 | 9.497944368 |
| Dnah8 | ENSMUSG00000033826 | 0.921989012 | 0.013715868 | 10.70688 | 11.06346 | 10.66315 | 11.31646298 | 11.57570495 | 12.19262915 |
| Rufy4 | ENSMUSG00000061815 | 1.702482634 | 0.01398916 | 6.904229 | 6.504051 | 6.36226 | 8.681196013 | 7.692766446 | 8.264899299 |
| CTGF | ENSMUSG00000019997 | -1.306315167 | 0.01444797 | 9.308277 | 9.228896 | 8.630472 | 7.999561225 | 7.890459666 | 7.436190012 |
| Sult1a1 | ENSMUSG00000030711 | -1.860493069 | 0.014821486 | 8.30963 | 7.360409 | 8.017342 | 6.380075534 | 6.239415674 | 5.74997089 |
| Wdfy1 | ENSMUSG00000073643 | -0.917276207 | 0.015867082 | 9.878942 | 10.4722 | 10.24758 | 9.253183399 | 9.352832162 | 9.314181754 |
| Csf1 | ENSMUSG00000014599 | 0.842404341 | 0.016528925 | 10.82442 | 11.66544 | 11.09907 | 12.0605681 | 11.94088959 | 12.22839256 |
| Clec5a | ENSMUSG00000029915 | 0.754924765 | 0.016528925 | 9.945224 | 10.04248 | 10.00326 | 10.93379817 | 10.6347443 | 10.6632195 |
| Adat2 | ENSMUSG00000019808 | 1.428178682 | 0.016528925 | 6.834198 | 7.658559 | 8.145945 | 9.092714639 | 9.010255097 | 9.058480298 |
| Il18 | ENSMUSG00000039217 | -1.620278714 | 0.016792748 | 8.325032 | 7.859395 | 8.528457 | 7.049524711 | 6.330088812 | 6.572483478 |
| Cd109 | ENSMUSG00000046186 | -2.315754848 | 0.016904708 | 8.590172 | 6.951545 | 7.028808 | 5.397105017 | 5.144277486 | 5.916376177 |
| Mmp8 | ENSMUSG00000005800 | 0.5778111 | 0.018824473 | 14.5964 | 14.68877 | 14.28695 | 15.08822907 | 15.06923193 | 15.17555346 |
| Igfbp5 | ENSMUSG00000026185 | -1.125196916 | 0.019192008 | 9.904797 | 11.04812 | 10.37796 | 9.675920959 | 9.397548625 | 9.06355793 |
| Alad | ENSMUSG00000028393 | -0.706570009 | 0.019330854 | 13.3218 | 12.72588 | 13.00835 | 12.25143306 | 12.53098862 | 12.19349794 |
| Ighv1-18 | ENSMUSG00000076695 | -1.826766272 | 0.019330854 | 9.206623 | 7.871065 | 9.83641 | 7.850688834 | 7.518632676 | 6.406570196 |
| Pkd1l3 | ENSMUSG00000048827 | -1.870361351 | 0.019330854 | 8.163079 | 8.215779 | 7.92858 | 6.134271846 | 5.400090579 | 6.935827062 |
| SEPP1 | ENSMUSG00000064373 | -0.707728378 | 0.019986372 | 12.84737 | 12.86187 | 13.04246 | 12.06226201 | 12.55891072 | 11.9403456 |
| Asprv1 | ENSMUSG00000033508 | 0.802436924 | 0.019986372 | 11.22277 | 11.91132 | 11.66008 | 12.72681397 | 12.32179958 | 12.17543581 |
| Ighv1-69 | ENSMUSG00000094502 | -2.046016779 | 0.022959107 | 7.934507 | 9.032437 | 9.412266 | 7.719435554 | 6.817618789 | 5.205452982 |
| Trf | ENSMUSG00000032554 | -0.649731805 | 0.023347393 | 13.51715 | 13.38004 | 13.66035 | 12.69615844 | 12.71154453 | 13.16436261 |
| Fbxl5 | ENSMUSG00000039753 | 0.666368372 | 0.024565083 | 12.76812 | 12.87841 | 12.32992 | 13.51741198 | 13.23204567 | 13.26408285 |
| Ighv2-3 | ENSMUSG00000094164 | -1.834837303 | 0.024565083 | 7.407035 | 7.479549 | 7.616829 | 5.837810003 | 5.806050309 | 5.590263828 |
| Flt4 | ENSMUSG00000020357 | -1.359134067 | 0.025884465 | 8.828547 | 10.13734 | 9.014273 | 7.881717684 | 8.205121604 | 8.201821727 |
| Nfkbiz | ENSMUSG00000035356 | 0.698608767 | 0.026276137 | 13.98335 | 14.40175 | 13.97444 | 15.10701954 | 14.47127649 | 14.84971092 |
| Sorl1 | ENSMUSG00000049313 | 0.633554251 | 0.027564334 | 14.16691 | 14.62532 | 14.16149 | 14.99632459 | 14.77070353 | 15.11673344 |
| Hcar2 | ENSMUSG00000045502 | 0.664663546 | 0.027564334 | 13.00707 | 13.0662 | 12.59394 | 13.53459738 | 13.34085892 | 13.79319702 |
| LOC105242706 | LOC105242706 | 0.883773468 | 0.0278853 | 11.12423 | 11.52752 | 10.57542 | 12.15471557 | 11.62444343 | 12.18449514 |
| C1qc | ENSMUSG00000036896 | -1.000080719 | 0.0278853 | 10.39289 | 10.37924 | 11.2062 | 9.960919263 | 9.791422305 | 9.329016672 |
| Col5a3 | ENSMUSG00000004098 | -1.454291563 | 0.0278853 | 10.58211 | 10.28533 | 9.160295 | 9.137109935 | 7.586419415 | 8.894347324 |
| Mov10 | ENSMUSG00000002227 | 0.932367229 | 0.0278853 | 9.52826 | 9.321121 | 9.215271 | 10.48202201 | 10.48492167 | 9.796711247 |
| Mmrn2 | ENSMUSG00000041445 | -1.206932055 | 0.0278853 | 9.519362 | 10.14217 | 9.212924 | 8.652696377 | 8.736254106 | 7.923281095 |
| Sdc1 | ENSMUSG00000020592 | -1.210891369 | 0.0278853 | 9.478626 | 9.389661 | 9.082717 | 8.497085047 | 8.228883437 | 7.474817527 |
| Pld3 | ENSMUSG00000003363 | -0.71547461 | 0.028871934 | 11.54119 | 11.78708 | 12.19372 | 11.10987979 | 11.20478239 | 11.13899624 |
| Aldh7a1 | ENSMUSG00000053644 | -1.059901358 | 0.028871934 | 9.487778 | 9.273548 | 9.297324 | 8.586436056 | 7.919953901 | 8.333764669 |
| Bst1 | ENSMUSG00000029082 | 0.621272807 | 0.030667292 | 10.86713 | 10.80439 | 10.66401 | 11.46573903 | 11.43314657 | 11.29877996 |
| Ccl3 | ENSMUSG00000000982 | 0.828813642 | 0.032672713 | 10.75221 | 10.47603 | 10.86574 | 11.96456283 | 11.34945294 | 11.16285032 |
| Ighv1-81 | ENSMUSG00000094689 | 0.830078469 | 0.032988319 | 10.00062 | 10.54868 | 10.05528 | 11.07271515 | 10.71937495 | 11.30361626 |
| Rps3a1 | ENSMUSG00000028081 | -0.6877138 | 0.034318063 | 12.68874 | 12.40286 | 12.59911 | 11.76392417 | 11.58953687 | 12.2159047 |
| Abcc3 | ENSMUSG00000020865 | -1.646325562 | 0.034472006 | 8.615444 | 8.578103 | 9.910705 | 7.77998123 | 7.993930865 | 6.469132058 |
| Il1rn | ENSMUSG00000026981 | 0.944426831 | 0.035774702 | 10.60752 | 11.34552 | 10.93145 | 11.68212278 | 11.43570129 | 12.47745345 |
| LOC102631780 | LOC102631780 | 1.159978062 | 0.035774702 | 8.394807 | 8.035659 | 8.273059 | 8.971032665 | 9.123604185 | 9.89825239 |
| Ddx60 | ENSMUSG00000037921 | 1.134973318 | 0.035881073 | 9.885882 | 9.403791 | 8.860813 | 9.918158052 | 10.47402648 | 11.0900712 |
| Igkv2-109 | ENSMUSG00000105606 | -2.44859622 | 0.035881073 | 6.382945 | 8.687752 | 7.566238 | 6.362585004 | 4.929070411 | 4.510873203 |
| Gdf15 | ENSMUSG00000038508 | -1.178678053 | 0.036590531 | 10.41465 | 10.36693 | 11.76434 | 9.743358153 | 9.477086325 | 10.18005059 |
| Sc5d | ENSMUSG00000032018 | 0.996012294 | 0.036590531 | 8.664698 | 9.282315 | 8.755083 | 10.06866597 | 9.512367508 | 10.10076535 |
| LOC105244211 | LOC105244211 | 0.847780908 | 0.03809919 | 10.44551 | 10.99378 | 10.1256 | 11.43364314 | 11.13292693 | 11.63012326 |
| Adam17 | ENSMUSG00000052593 | 0.620677918 | 0.03900821 | 12.3324 | 12.72206 | 12.53737 | 13.03181154 | 12.99817028 | 13.41174765 |
| F11r | ENSMUSG00000038235 | -1.109561554 | 0.040734053 | 9.048609 | 9.64889 | 9.20113 | 8.50108714 | 8.288892669 | 7.794830008 |
| Nfib | ENSMUSG00000008575 | -2.262101412 | 0.041548672 | 7.107899 | 7.787318 | 6.491824 | 5.648724281 | 4.560931382 | 4.876669547 |
| Igkc | ENSMUSG00000076609 | -0.831426369 | 0.043642871 | 14.27643 | 14.55091 | 14.94455 | 14.15749507 | 13.90932292 | 13.08808442 |
| Pirb | ENSMUSG00000058818 | 0.544310262 | 0.043642871 | 12.88444 | 12.91198 | 12.68926 | 13.55579518 | 13.310558 | 13.24241755 |
| Ighv1-72 | ENSMUSG00000096074 | -1.855920817 | 0.043642871 | 7.377795 | 9.389661 | 8.203827 | 7.165014239 | 6.902807886 | 5.870757626 |
| Lepr | ENSMUSG00000057722 | -1.173915816 | 0.045481306 | 10.30631 | 10.91493 | 10.39359 | 10.10198649 | 9.100605655 | 8.540912716 |
| Marcksl1 | ENSMUSG00000047945 | 0.663492638 | 0.046593323 | 11.7913 | 12.28976 | 11.98053 | 12.87700057 | 12.38158704 | 12.78987434 |
| Tagap | ENSMUSG00000033450 | 0.59702807 | 0.046821766 | 11.28905 | 11.12141 | 11.08116 | 11.71952986 | 11.61770936 | 11.93273471 |
| Tfpi | ENSMUSG00000027082 | -0.81794416 | 0.04754199 | 9.91501 | 10.18604 | 9.839457 | 9.144804527 | 9.012994838 | 9.345786154 |
| Nlrp3 | ENSMUSG00000032691 | 0.613122719 | 0.048089891 | 13.51029 | 13.87784 | 13.50463 | 14.43796206 | 13.9371763 | 14.34181287 |
| LOC105244302 | LOC105244302 | 2.364771541 | 0.048158082 | 3.940248 | 5.281486 | 4.765032 | 6.559654742 | 6.854746004 | 7.330632731 |
| Iglv1 | ENSMUSG00000076934 | -0.915873788 | 0.048201584 | 10.8645 | 11.09117 | 11.55234 | 10.09802843 | 10.76965158 | 9.814891838 |
| Igkv5-43 | ENSMUSG00000094433 | -1.460443776 | 0.049602649 | 8.978919 | 9.781572 | 8.751853 | 8.295241986 | 7.971570064 | 6.682188969 |

Supplementary Figure 1. Confirmation of endosteal cell removal following collagenase treatment. Endocortical surfaces were processed for histology and stained with H&E to assess cell removal. PBS-treated samples showed residual cells along the endocortical surface (blue arrow), whereas collagenase-treated samples exhibited few to no remaining cells, confirming effective removal.

**Supplementary Figure 2. Negative control for immunofluorescence analysis.** Negative control for the immunofluorescence analyses was obtained following the reported protocol, but without incubation with the primary antibody (anti-Paxillin) to confirm signal specificity.

**Supplementary Figure 3. Conditional deletion of Wnt5a in osteoclast precursors affects femoral biomechanical properties in a sex-specific manner. (A)** Torsion biomechanical testing of femurs revealed significant reduction in maximum torque and work to failure in male *Wnt5a* cKO compared to controls, while females were unaffected by the mutation. Statistical analysis was performed by unpaired, two-tailed *t*-test (males n = 11-12/genotype; females n = 9-13/genotype). * p < 0.05, ** p < 0.01.

**Supplementary Figure 4.** Gene ontology (GO) analysis for biological process **(D)**, molecular function **(E)**, and cellular component **(F)** using differential expression genes (DEGs) in *Wnt5a* cKO cells. Y-axis label represents gene ontology (GO) terms, and X-axis represents GeneRatio (the ratio of genes annotated to a given GO term to the total number of genes in the DEG set). The size of the bubble represents the gene counts enriched in a particular GO term and the color indicates the false discovery rate (FDR) of GO terms.

**Supplementary Figure 5.** Picro Sirius Red staining of collagen fibers in femur sections revealed no significant changes in females, with a trend towards a reduction in the percentage of red-orange fibers (n = 4-5/genotype). Statistical analysis was performed by unpaired, two-tailed t-test. * p < 0.05.
